# Single-cell phenotyping of extracellular electron transfer via microdroplet encapsulation

**DOI:** 10.1128/aem.02465-24

**Published:** 2025-01-14

**Authors:** Gina Partipilo, Emily K. Bowman, Emma J. Palmer, Yang Gao, Rodney S. Ridley, Hal S. Alper, Benjamin K. Keitz

**Affiliations:** 1 McKetta Department of Chemical Engineering, The University of Texas at Austin 530061 , Austin, Texas, USA; 2 Interdisciplinary Life Sciences Graduate Program, The University of Texas at Austin 12330 , Austin, Texas, USA; 3 Civil, Architectural, and Environmental Engineering, The University of Texas at Austin 439290 , Austin, Texas, USA; Danmarks Tekniske Universitet The Novo Nordisk Foundation Center for Biosustainability, Kgs. Lyngby, Denmark

**Keywords:** microbiology, microfluidics, electroactive, iron-reducing

## Abstract

Electroactive organisms contribute to metal cycling, pollutant removal, and other redox-driven environmental processes via extracellular electron transfer (EET). Unfortunately, developing genotype-phenotype relationships for electroactive organisms is challenging because EET is necessarily removed from the cell of origin. Microdroplet emulsions, which encapsulate individual cells in aqueous droplets, have been used to study a variety of extracellular phenotypes but have not been applied to investigate EET. Here, we describe the development of a microdroplet emulsion system to sort and enrich EET-capable organisms from complex populations. We validated our system using the model electrogen *Shewanella oneidensis* and described the tooling of a benchtop microfluidic system for oxygen-limited conditions. We demonstrated the enrichment of strains exhibiting electroactive phenotypes from mixed wild-type and EET-deficient populations. As a proof-of-concept application, we collected samples from iron sedimentation in Town Lake (Austin, TX) and subjected them to microdroplet enrichment. We measured an increase in electroactive organisms in the sorted population that was distinct compared to a population growing in bulk culture with Fe(III) as the sole electron acceptor. Finally, two bacterial species not previously shown to be EET-capable, *Cronobacter sakazakii* and *Vagococcus fessus*, were further cultured and characterized for electroactivity. Our results demonstrate the utility of microdroplet emulsions for isolating and identifying EET-capable bacteria.

**IMPORTANCE:**

This work outlines a new high-throughput method for identifying electroactive bacteria from mixed populations. Electroactive bacteria play key roles in iron trafficking, soil remediation, and pollutant degradation. Many existing methods for identifying electroactive bacteria are coupled to microbial growth and fitness—as a result, the contributions from weak or poor-growing electrogens are often muted. However, extracellular electron transfer (EET) has historically been difficult to study in high-throughput in a mixed population since extracellular reduction is challenging to trace back to the parent cell and there are no suitable fluorescent readouts for EET. Our method circumvents these challenges by utilizing an aqueous microdroplet emulsion wherein a single cell is statistically isolated in a pico- to nano-liter-sized droplet. Then, via fluorescence obtained from copper reduction, the mixed population can be fluorescently sorted and gated by performance. Utilizing our technique, we characterize two previously unrecognized weak electrogens *Vagococcus fessus* and *Cronobacter sakazakii*.

## INTRODUCTION

In the absence of oxygen, electroactive microorganisms transfer electrons in/out of the cell to oxidize/reduce extracellular soluble and insoluble metal species in a process known as extracellular electron transfer (EET) (1). This process is coupled to microbial growth, respiration, communication, and sensing (2). EET has been implicated in metal transport (1), environmental remediation (3–6), human health (7–10), and more. Furthermore, EET has been co-opted for the treatment of wastewater (11, 12), power generation in microbial fuel cells (13–18), and biocatalysis (19–25). Electroactive bacteria have been isolated from a variety of locations including in aquatic and soil environments (11, 26–28), the human gut microbiome (7–9, 29), and oral biofilms (30). Traditional methods for identifying EET-capable microbes involve competitive respiration on poised iron or graphite electrodes, or other metal-based electron acceptors (11, 31–40). Unfortunately, these techniques make it difficult to establish genotype-phenotype relationships, especially in complex consortia, because EET occurs in the extracellular space. Bulk enrichments using poised electrodes partially avoid this challenge by selecting biofilms (15) comprised of a single or a handful of different electroactive species, but these experiments may not capture the complexity of the initial isolate (32, 40–43). In addition, a single species that forms a stable biofilm in bioelectrochemical cells can prevent other species from being incorporated into the biofilm altogether (42, 44). These low-throughput, bulk enrichment strategies require successful candidates to outcompete other microbes for nutrients, electron acceptors, carbon sources, and access to the electrode. They also require a microbe to be culturable in a laboratory environment. As a result, bulk enrichment does not typically allow for more nuanced analysis of individual bacteria or the identification of EET in less fit, lower relative abundance, and unculturable microorganisms (Table 1). Furthermore, while computation methods (such as FeGenie (45)) can link EET behavior to specific protein pathways, these tools rely on previously characterized pathways which may not recognize weak or new electrogenic behavior. Therefore, there is a need for high-throughput identification of single-cell EET behavior to identify novel electrogens in complex environments (33, 46, 47).

**TABLE 1:** Current methods for enriching EET-capable organisms from a mixed population

Method	Time	Throughput	Drawbacks
Microbial fuel cells (42, 48)	5–20 days	Low (1–2 isolates)	Biofilm formation, growth competition
Growth on soluble Fe(III) (49)	5–14 days	Medium (2 + isolates)	Growth competition, false positives
Microdroplet emulsion microfluidics (this work)	3 days	High (assessment of 7,000–10,000 filled droplets)	Benchtop anaerobicity

Cytometric techniques, such as flow cytometry and flow-assisted cell sorting (FACS), are powerful tools for characterizing complex populations but require intracellular reporters and cannot be easily translated into extracellular processes. Extracellular phenotypes including cell surface display, secretion, and other processes (50) can be characterized using microdroplet emulsions, which encapsulate cells in pico- to nano-liter droplets and can be sorted similarly to FACS. Droplet creation consists of emulsifying a dilute aqueous solution containing microbes with a counter-current oil flow. Aqueous droplets can then be cultured, merged with fresh aqueous solutions, and analyzed and sorted via fluorescence. Essentially, single-cell aqueous microdroplets function as individual reaction wells, capturing extracellular products or fluorescence (51) and muting cell-to-cell competition. Previously, microdroplet screening has been used for bioprospecting phenotypes of interest from environmental populations (52–54), but to the best of our knowledge has not been used to identify electroactive organisms.

Here, we describe the development of a microdroplet encapsulation assay for characterizing and sorting electroactive bacteria. Our assay leverages a previously reported Cu(I)-catalyzed alkyne-azide cycloaddition (CuAAC) that generates a fluorescent product (55) in response to EET activity (23). We developed our system using a monoculture of the model electroactive organism *Shewanella oneidensis* MR-1. A mixed population of EET-deficient strains and EET-capable strains of *S. oneidensis* facilitated fluorescent activated droplet sorting (FADS) to enrich EET-capable strains from mixed populations. As an application, we identified EET-capable microbes from an environmental sample. We measured a distinctly enriched EET-capable population, which had upregulation of iron reduction genes compared to the initial consortium. Two bacteria, *Cronobacter sakazakii* and *Vagococcus fessus*, identified by our microdroplet screen were assessed in monoculture and displayed marked electrogenic behavior. In summary, we describe the development of a non-growth-related, high-throughput screen for identifying extracellular reduction by bacteria.

## RESULTS

### Optimization of CuAAC fluorescent probe assay for applications in microdroplets

**Fig 1:**
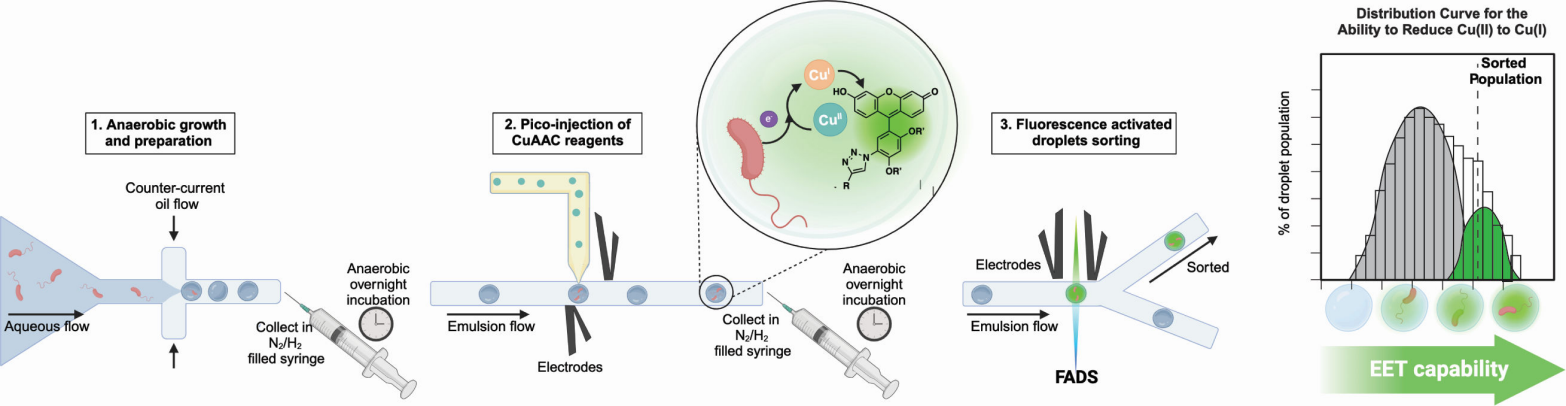
Microdroplet assay setup and schematic. The reaction scheme for performing oxygen-limited CuAAC via microbial respiration in a microfluidic system. Inset is an image of *S. oneidensis*-containing microdroplets image prior to sorting. Briefly, on the first day, a dilute aqueous solution of cells is used to create an emulsion via counter-current flow with a perfluorinated oil. The emulsion is collected into an oxygen-free syringe and incubated overnight. On the second day, the emulsion flowed past an aqueous stream containing the CuAAC reagents. A low voltage (0.15 V, indicated by electrodes spanning both sides of the channel) is applied to merge the aqueous stream into the existing emulsion. The emulsion is collected into an oxygen-free syringe and incubated overnight. Finally, on the third day, the emulsion is opened to oxygen and flowed by a laser which excites the fluorophore in the emulsion. Using a photomultiplier tube (PMT), the fluorescence is detected and a low voltage (0.25–0.35V, indicated by electrodes flanking the laser lines) can be applied to pull fluorescent droplets into the sorted channel. The remaining droplets are left unsorted to flow down the channel. Further details are outlined in Materials and Methods and Fig. S3.

We recently utilized fluorescence from the EET-driven synthesis of a cycloaddition probe, CalFluor488 (55), to assess EET activity from *S. oneidensis* via Cu(I)-catalyzed alkyne-azide cycloaddition (CuAAC) (23). The CalFluor488 probe undergoes a quenched-to-fluorescent conversion upon the creation of the triazole with an increase in fluorescence over 200-fold (23). We previously determined that the rate of copper reduction was directly correlated to EET flux via well-defined EET-protein pathways in *S. oneidensis* (the Mtr-pathway) and that fluorescence was tied to electron transfer. As a result, we hypothesized that the fluorescence readout from this reaction could be adapted into a screen for identifying electroactive microbes in microfluidic droplets. The quenched-to-fluorescent transition is ideal for microfluidic applications because the unreacted emulsion has little background fluorescence (Fig. 1; Fig. S2). Similar to FACS, a photomultiplier tube (PMT) can convert small fluorescent signals to quantifiable readouts where fluorescence inside a droplet correlates to a higher voltage.

First, we confirmed that neither the reaction nor the fluorescence output was inhibited by the reagents required for a stable microdroplet emulsion. Traditionally, a rich media without organic solvents is required for stabilizing emulsions and supporting microbial growth within droplets (50). Our previous use of *S. oneidensis* to perform CuAAC was performed in minimal media with DMSO as a co-solvent (23), but these conditions yielded droplet instability in initial screens. Anaerobic reactions performed with *S. oneidensis* in a 96-well plate showed that nearly all DMSO could be omitted apart from the small volume of co-solvent in the CalFluor488 stock. Neither cell growth nor conversion was hindered under these conditions even with increased concentrations of reagents (Fig. S1). Finally, the reaction was not significantly inhibited by the presence of fluorinated oil (with or without the biosurfactant present) and we measured a large fluorescence response in well-plate controls (Fig. S1).

Having confirmed that the reagents necessary for a stable emulsion did not interfere with cell growth or CuAAC activity, we adapted this assay to the microdroplet system. Abiotic emulsions were created with starting material and chemically synthesized products. The emulsions were mixed in known ratios and analyzed via FADS to measure our ability to distinguish the reacted product from the background (Fig. S2). As expected, we detected differences in the emulsion populations including at the target level of cellular encapsulation (one fluorescent droplet per every 10 droplets). Together, these benchtop assays suggested that EET-driven CuAAC could be utilized as a microbially driven fluorescent readout for microdroplet emulsion sorting.

### Oxygen-limited benchtop microdroplet system enabled Cu(I)-catalyzed alkyne-azide cycloaddition (CuAAC) within microdroplets

Wild-type *S. oneidensis* can convert CalFluor488, to a fluorescent cycloaddition product in approximately 5 h when combined and sealed with the appropriate Cu-catalyst and alkyne source (23). However, we found that an emulsion created from an aerobic culture of *S. oneidensis* (OD_600_ of 6 × 10^−5^, calculated such that one in every 10 droplets was filled (56)) did not yield the fluorescent signal above the background after 24 h following encapsulation with the CuAAC reagents. In addition, no *S. oneidensis* was recovered after breaking the emulsions and plating on agar for recovery. Hypothesizing that the dilute cells could not withstand the simultaneous stress of encapsulation in the presence of Cu(II/I), small molecules, and shift to anaerobic metabolism, we adapted a previously developed method for injecting biosensors in microdroplets (50).

**Fig 2:**
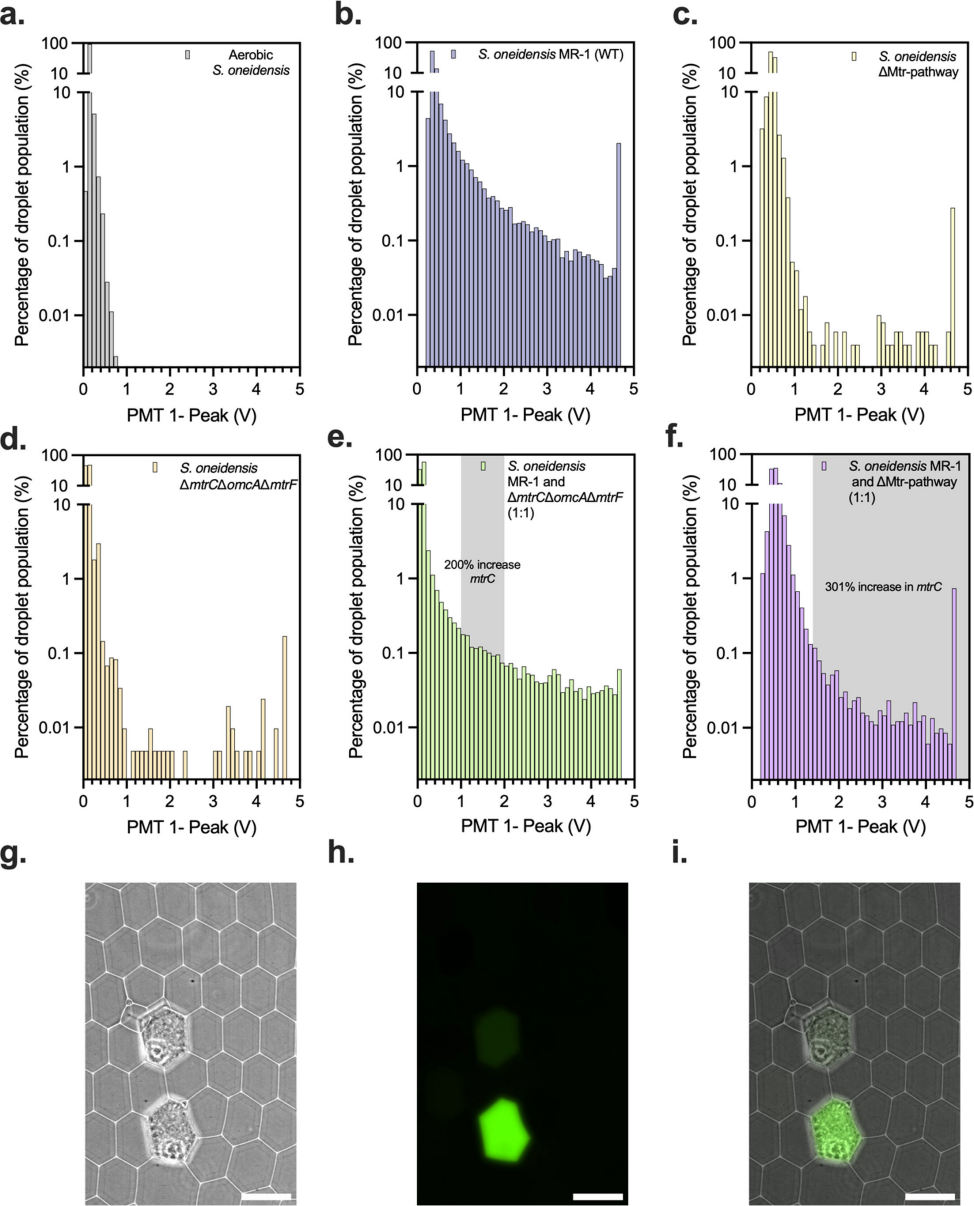
Oxygen-limited conditions allow for detection using of *S. oneidensis* in microdroplet system. (a) Histogram of aerobic FADS of *S. oneidensis.* (b) Histogram of an oxygen-limited FADS of *S. oneidensis*. (c) Histogram of an oxygen-limited FADS of ∆Mtr-pathway *S. oneidensis.* (d) Histogram of an oxygen-limited FADS of ∆*mtrC*∆*omcA*∆*mtrF S. oneidensis*. (e) Histogram of an oxygen-limited FADS of *S. oneidensis* wild-type and *mtrC*∆*omcA*∆*mtrF S. oneidensis* mixed in a 1:1 ratio prior to encapsulation. (f) Histogram of an oxygen-limited FADS of *S. oneidensis* wild-type and *S. oneidensis* ∆Mtr-pathway mixed in a 1:1 ratio prior to encapsulation. (g–i) Representative image of emulsion from panel f prior to sorting with bright field (g), fluorescence (h), and merged bright field and fluorescence (i). The scale bar represents 100 µm.

Specifically, we utilized “pico-injection,” which flows a previously formed emulsion through a microfluidic chip and introduces a fresh aqueous solution into each droplet by applying a low voltage to merge the emulsion with the injected solution (Fig. 1). Previously, microdroplet emulsions have been pico-injected to add cell-based biosensors (50), cell lysis reagents (57, 58), or fluorogenic enzyme substrates (59–62) into existing emulsions. We hypothesized this mechanism would provide robustness by allowing a recovery period post-encapsulation before withstanding the stress of the Cu(II/I) and small molecule reagents (Fig. 1; Fig. S3). We moved to pico-inject the CalFluor488, Cu-catalyst, and alkyne after emulsification and stabilization for a full day. The 5× concentrated pico-injection solution was flowed into the emulsion utilizing a Pico-Mix chip (Sphere Fluidics) and merged under a low voltage of 0.15V. This facilitated growth within the droplets (Video S1). We measured no detectable fluorescent signal even under the pico-injection scheme (Fig. 2a); however, we were able to recover cells when plated on agar plates. We posited that the emulsion was oxygen-permeable, and the oxygen was inhibiting the CuAAC reaction and preventing a complete shift to anaerobic metabolism in *S. oneidensis*.

To overcome this challenge, we performed subsequent experiments in an oxygen-limited environment. All oils and buffers were sparged and prepared anaerobically. Tubing was attached, and the syringes were sealed within an anaerobic chamber to create a closed environment. A 12 mL collection syringe was prepared with a small, cushion of fluorinated oil (500 µL), and an anaerobic atmosphere (6 mL of 97% N_2_, 3% H_2_), then sealed (Fig. S3). The solutions and oils were removed from the chamber and loaded onto the syringe pumps. To prevent oxygen from entering the system, for both droplet encapsulation and pico-injection, a positive pressure was applied to each syringe before removing the seal and plumbing the microfluidic lines into the chip (Fig. S3). Once the system had equilibrated and the emulsion or pico-injection was stable in size, the collection syringe could be used to collect the emulsion. As predicted, this oxygen-limited setup allowed *S. oneidensis* MR-1 encapsulated within droplets to perform CuAAC, which could be detected using our system (Fig. 1 and 2b). The fluorescence in the emulsion was also visible using fluorescent microscopy and the ratio of fluorescent to non-fluorescent droplets approximated the predicted encapsulation ratio, one out of every 10 droplets filled. Using the FADS system, we detected fluorescence in a higher percentage of the droplet population relative to chemical controls lacking cells (Fig. 2b). In total, these results indicate that the bacteria can perform sufficient EET within the microdroplet system to catalyze CuAAC and that extracellular reduction is detectable via fluorescence under anaerobic conditions (Fig. 2b).

### CuAAC for the detection of extracellular electron transfer in microdroplets

Next, to confirm that the signal was tied to EET, homogeneous populations of two different EET-deficient strains of *S. oneidensis* (ΔMtr and ∆*mtrC*∆*mtrF*∆*omcA*) were encapsulated and characterized by FADS. As expected, both monocultures generated significantly less signal than the wild-type *S. oneidensis* (Fig. 2c and d). To confirm the cells survived droplet encapsulation and pico-injection, we subjected cells to FADS and broke the emulsion to harvest the cells. Lawns were recovered after plating broken emulsions from non-fluorescent droplets, indicating that the lack of fluorescent response was due to deficiencies in EET, not from disruptions in cell viability (Fig. S4). Having demonstrated that CuAAC within microdroplets could distinguish between homogeneous populations in individual emulsions, we next examined a mixed culture of wild-type and EET-deficient strains of *S. oneidensis* (Fig. 2e and f).

A 1:1 ratio of wild-type *S. oneidensis* to an ∆Mtr-pathway knockout strain (EET-deficient) was mixed immediately prior to encapsulation. Prior to FADS, microscopy revealed a sample with 181 droplets containing bacteria. Of those, 75 droplets also fluoresced (Fig. 2i), which roughly approximated the 1:1 starting encapsulation ratio. The difference in fluorescent signal indicated that the ∆Mtr-pathway strain could be differentiated from wild-type *S. oneidensis* by fluorescence and the sample was sorted via FADS. A sample of the emulsion sorted above 1.4 V, was broken, plated, and subjected to a colony PCR for *mtrC*. In the unsorted population (*n* = 34), we determined that 19% of colonies contained *mtrC*, while in the sorted population (*n* = 36) 78% of colonies contained *mtrC*, representing an approximately 300% increase (Fig. S5). An additional emulsion containing a 1:1 ratio of wild-type *S. oneidensis* to ∆*mtrC*∆*mtrF*∆*omcA* was sorted and a subsection of the high-fluorescence emulsion was collected, broken, and plated on agar plates. From the sample collected, a colony PCR for the *mtrC* gene was performed on the sorted and unsorted populations (*n* = 40) revealing a 200% enrichment of the *mtrC*-containing colonies in the sorted population (67.5% versus 22.5%) (Fig. S5). These results validated that the microdroplet system could enrich EET-active strains in a mixed population.

### Single-cell analysis of environmental samples reveals EET-capable bacteria

**Fig 3:**
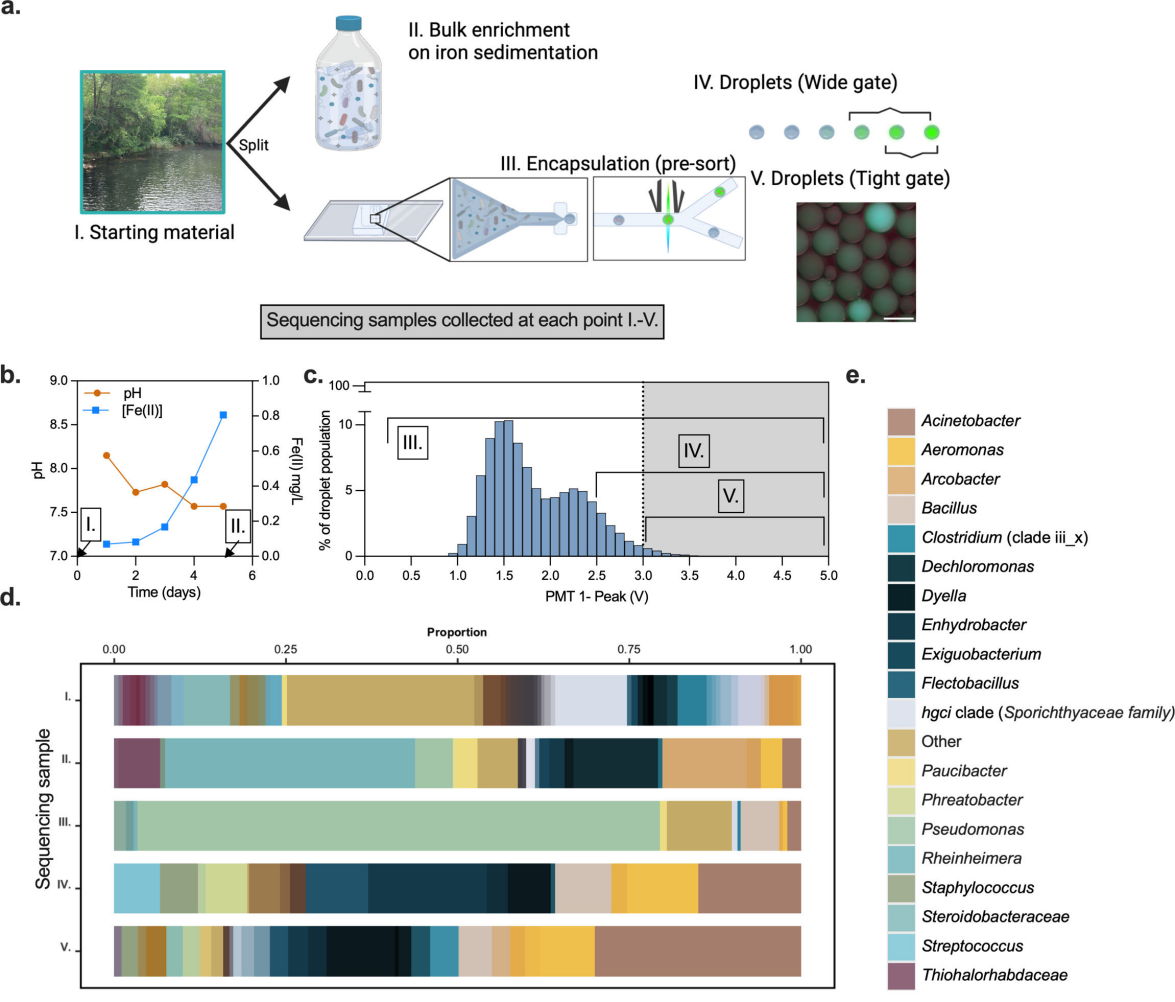
Iron sediment gathered from Town Lake yields Fe(III)-reducing bacteria. (a) A schematic outlining the experimental procedure, and where samples were gathered for 16S sequencing. Briefly, bacteria were collected from iron sedimentation. This starting material was sequenced and split into a bulk enrichment and a microdroplet enrichment. The microdroplet samples were sequenced pre- and post-sorting. Two different gates of the sorted enrichment were collected and subjected to sequencing. The inset of droplets is a representative image of the lake water emulsion prior to sorting, and the scale bar represents 100 µm. Points I–V indicate when sequencing samples were taken. (b) Bulk enrichment data monitoring pH and Fe(II) concentration over time. Points I and II indicate when sequencing samples were taken. (c) Histogram of microdroplet emulsion and gating of samples IV and V. Histogram represents a relative proportion of population after the system has stabilized and collected for 87 k droplets. Sorted populations were collected for a minimum of 500 droplets collected. Points III–V indicate when sequencing samples were taken. (d) Relative genus distributions of samples with greater than 0.05% prevalence. (e) Top 20 genera as described in d.

Next, compared the microdroplet enrichment to a more traditional, bulk enrichment for EET using a sediment-associated mixed microbial community collected from Town Lake in Austin, TX (30°16′25.2″N, 97°46′13.4″W). To benchmark the microdroplet system, part of the sample was enriched via anaerobic growth with a solid-phase Fe(III) source to select for electroactive, EET-capable microbes. This bulk enrichment experiment included lactate as a carbon source and iron-rich sediment as the electron acceptor. Sterilized sediments were isolated within a 3.5 kDa MWCO dialysis membrane tube to limit the contact between bacteria and iron minerals and biofilm formation (49, 61, 63, 64). The bulk enrichment culture was monitored for both pH and soluble Fe(II) concentration over time (Fig. 3b) and displayed an increase in soluble Fe(II) from 0.069 to 0.8 mg/mL over 5 days indicating that this biological sample contained EET-capable organisms. Samples from before and after enrichment were harvested and subjected to 16S sequencing (Fig. 3, samples I and II).

The other aliquot of sediment-derived sample was subjected to microfluidic FADS, where a clear sub-population exhibited improved fluorescence (Fig. 3a), as indicated by a tailing end of high performers (Fig. 3c). To evaluate the effect of microdroplet formation, both the initial sediment-derived population (Sample I) and the population immediately prior to sorting (Sample III) were both collected for 16S sequencing. To evaluate the effect of sorting the population by fluorescence, two portions of the population were sorted out, wide gated (greater than 2.5 V) (Sample IV) and tight gated (greater than 3.1V) (Sample V) and collected for 16S sequencing.

### 16S sequencing allowed for the identification of high-performing targets

**Fig 4:**
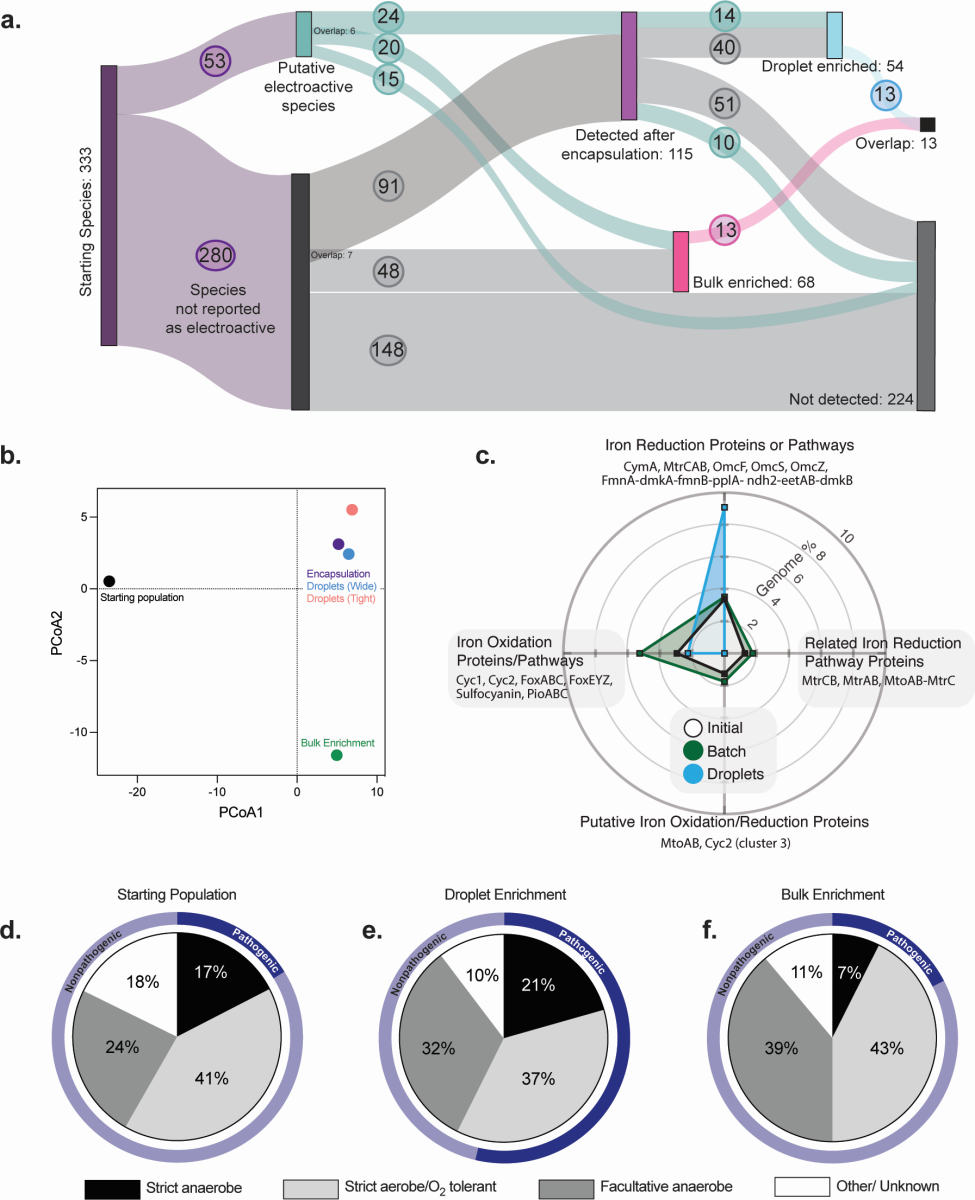
Analysis of species similarity between enrichment mechanisms. (a) Sankey diagram describing the species distribution of the initial lake water and enrichment techniques. Line overlap right of putative electroactive species and not-reported electroactive species indicates that species were found in both samples. (b) Principal component analysis of the genus data represented in Fig. 3d with a *k* = 2. (c) Percent of species containing one or more genes related to iron-regulation pathways as determined via HMM with FeGenie (45). (d–f) Venn diagram describing the relative species metabolism distributions and pathogenicity.

The 16S sequencing data, collected from the bulk and droplet enrichments, were normalized by the number of reads per sample, and compared to the starting, untreated lake water. From the bulk enrichment (Sample II), 68 bacteria were identified and 20 (29%) of these had been previously thought to be capable of EET (27, 32, 38, 46, 65–68) (Fig. 4a). The remaining 48 could indicate “cheaters,” bacteria that survived in bulk but were not actively reducing iron upon harvest or previously unknown electroactive bacteria. The bulk enrichment favored the *Rheinheimera*, *Dechloromonas*, and *Arcobacter* genera (Fig. 3d). In the microdroplet system, when compared to the initial lake water, 45 species were enriched by encapsulation alone (Sample III). A significant portion of the population that was present in the unsorted microdroplets screen were members of the *Pseudomonas* genus (Fig. 3d). However, the sorted droplet populations (Samples IV and V) were enriched and favored *Exiguobacterium*, *Acinetobacter*, and *Aeromonas* genera, indicating that the sorting selects for reduction as opposed to selecting exclusively for survival within droplets. We identified a total of 54 bacterial species in the fluorescently sorted droplets, suggesting some ability to reduce Cu(II) to Cu(I). Of the bacterial species identified in the droplet-sorted population, 14 (26%) had been previously reported as potentially EET-capable (27, 32, 38, 46, 65–68). However, of the 333 species of bacteria detected in the starting material (or in a subsequent enrichment), only 53 (16% of the starting material) were previously proposed as being EET-capable bacteria and, only 24 (45% of the previously reported) of those were detected after encapsulation. This indicated that some were either unable to withstand the sediment-to-microdroplet transition or did not survive the initial reconstitution from iron sedimentation. Given that only 24 putative EET-capable species were detected after encapsulation, 14 (58%) of the putative EET bacteria within the pre-sort droplets were identified by our screen indicating that the screen was effective for detecting EET activity (Fig. 4a).

Interestingly, when comparing the microdroplet enrichment (Samples IV and V) and bulk enrichment (Sample II), only 13 bacteria were enriched under both conditions. Only six of these shared species had been previously referenced as EET-capable: *Acinobacter johnsonii, Aeromonas salmonicida, Aeromonas veronii*, *Ralstonia solanacearum,* an unidentified *Aeromonas* species and a *Citrobacter* species. Seven other species were identified by both methods but had never been characterized as having the ability to perform EET (Fig. 4b). A principal component analysis (*k* = 2) revealed that the starting material, droplets, and bulk enrichment were clustered separately (Fig. 4b), indicating that the populations of these samples were distinct. Finally, rarefaction curves (Fig. S6) suggested a drop in diversity between the starting material and the encapsulation, as well as the encapsulation and the sorted samples indicating that there was a bottleneck that decreased sample diversity. Together, the 16S sequencing data indicate that we enriched for a distinct sub-section of the population in the microdroplet FADS that was different from the bulk enriched population and potentially represents a phenotype with the ability to perform EET.

### FeGenie analysis of sorted populations reveals differing enrichments

To further probe the features of the different enrichments, example genomes from each enriched bacteria (for which a fully sequenced genome was available) were analyzed using FeGenie to identify iron-trafficking genes (45). FeGenie looks for homology using a Hidden Markov Model (HMM) against known iron-related protein pathways. We used it to identify whether a given bacterium within a population has one or more genes related to a known iron-redox pathway. Looking at four of the gene clusters related to iron reduction, the microdroplet system enriched for bacteria containing known pathways involved in iron reduction (CymA, MtrCAB, OmcF, OmcS, OmcZ, FmnA-dmkA-fmnB-pplA-ndh2-eetAB-dmkB (46, 69–72)), notably including those most similar to what is found in *S. oneidensis* (1, 73–76). Compared to the initial consortium, 2.7 times more bacteria had one or more genes related to this specific iron reduction pathway, nearly 10% of the genomes analyzed (Fig. 4a). The program also screens for genes related to iron reduction pathways where there is no homolog to the terminal *mtrC*. These are characterized as “related to iron reduction” because they represent a potential, but incomplete pathway (45, 69–72). These bacteria containing “related to iron reduction” genes (MtrCB, MtrAB, and MtoAB-MtrC) were enriched over 1.4-fold relative to initial consortia in the bulk enrichment. However, given that the mechanisms of EET are diverse, these data only capture genes prevalent in previously characterized electrogens. Interestingly, in the bulk enrichment, we measured an increase in the presence of genomes containing one or more genes related to iron oxidation. This was likely due to the reduction of Fe(III) to Fe(II) in the bulk enrichment, which could be used for iron oxidation by other bacteria. Combined, these data suggest competing mechanisms of enrichment that likely may contribute to the low number of shared bacteria between the enrichment techniques (Fig. 4a). In addition, our results indicate that the microdroplet enrichment sorted for a distinct population of electrogens compared to the bulk enrichment, which may select for interspecies collaborations or sub-sets of EET-mechanisms that represent multiple simultaneous phenotypes.

### Monoculture characterization of high-performers from microdroplet enrichment reveals putative electrogens

**Fig 5:**
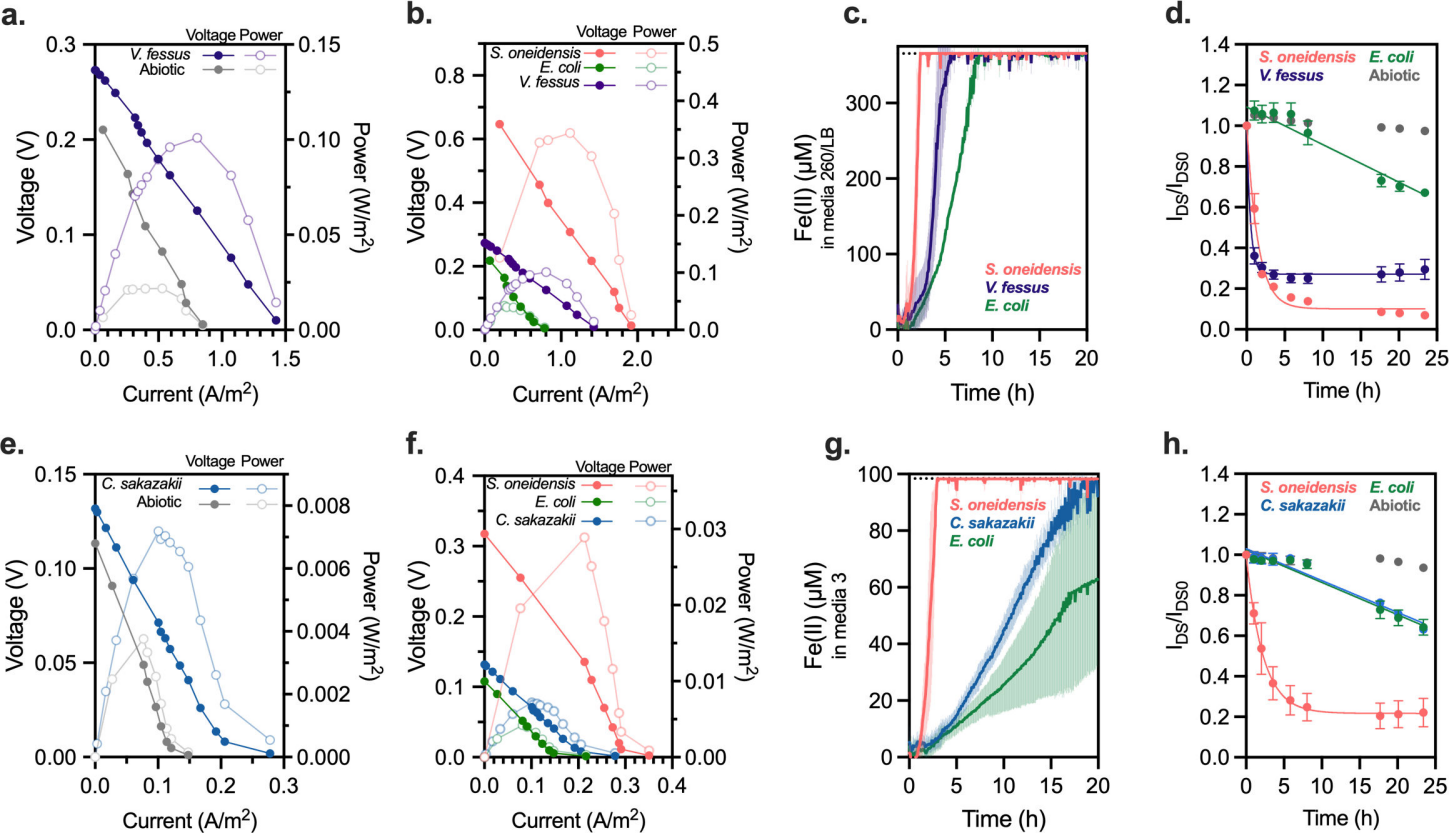
Examination of putative electrogens *C. sakazakii* and *V. fessus*. (a) MFCs to measure voltage and power generation of *V. fessus* and Media 260. (b) MFCs to measure voltage and power generation of *S. oneidensis* and *E. coli* in Media 260 compared to *V. fessus* in Media 260 (*V. fessus* curves re-printed from a. for comparison purposes). (c) Fe(II) generation curves measured via ferrozine colorimetric assay over time. The initial inoculum of OD_600_ = 0.01 was from the same bacterial stock of *V. fessus* used to generate (d) and run in the same respective media. (d) Current over time from bacterially generated de-doping of a PEDOT:PSS-coated electrode. The I_DS_/I_DS0_ curve was prepared with an initial inoculum OD_600_ = 0.01 in a 1:50 mixture of Media 260:LB broth. (e) MFCs to measure voltage and power generation of *C. sakazakii* and Media 3. (f) MFCs to measure voltage and power generation of *S. oneidensis* and *E. coli* in Media 3 compared to *C. sakazakii* in Media 3 (*C. sakazakii* curves re-printed from e. for comparison purposes). (g) Fe(II) generation curves measured via ferrozine colorimetric assay over time. The initial inoculum of OD_600_ = 0.01 was from the same bacterial stock of *C. sakazakii* used to generate (h) and run in the same respective media. (h) Current over time from bacterially generated de-doping of a PEDOT:PSS-coated electrode. The I_DS_/I_DS0_ curve was prepared with an initial inoculum OD_600_ = 0.01 Media 3.

Finally, we determined whether select species isolated in the highest performing droplet sorts were EET-capable or if we were enriching for alternative phenotypes. Due to the destructive nature of 16S sequencing, we required species that were commercially available and laboratory-culturable. Two bacteria, *Cronobacter sakazakii* and *Vagococcus fessus,* fit these criteria and were enriched solely in the highest sort (tight sort, Sample IV) gating of the lake water microdroplet enrichment. Neither bacterium was enriched in the bulk enrichment nor had they previously been reported as EET-capable, although *C. sakazakii* has been known to have iron-transport and acquisition-related machinery that is vital to its survival (77, 78). Each bacterium was grown in its preferred culture media and as benchmarks, *E. coli* MG1655 and *S. oneidensis* MR-1 were measured concurrently in each respective media and evaluated for their EET-activity. *S. oneidensis* was chosen as a strong electrogen and *E. coli* was chosen due to its latent EET-metabolism representing a weak electrogen (79). A traditional method of identifying EET-capable bacteria, microbial fuel cells (MFCs), was used to create polarization curves for each species in its respective media (Fig. 5a, b, e, and f) once stabilization had occurred. Broadly, *S. oneidensis* yielded a high power output, *V. fessus* and *C. sakazakii* achieved moderate output, and *E. coli* barely measured an appreciable increase over the background. Next, to determine their ability to reduce soluble Fe(III), a Fe(III/II) reduction assay and growth on Fe(III) was collected for each bacteria. Both bacteria reduced Fe(III) to Fe(II) over 20 h as measured by the colorimetric ferrozine assay (80) (Fig. 5c and g; Fig. S8). Neither bacteria appeared to grow with Fe(III) as the sole electron acceptor (Fig. S8); however, we were unable to establish culturing conditions for *V. fessus* without the presence of at least 0.1% sheep’s blood (Fig. 5e and f; Fig. S7). Finally, the bacteria were incubated in an organic electrochemical transistor (OECT) under continuous electrode bias conditions to further examine their ability to reduce insoluble electron acceptors. OECTs, which have a faster response and require smaller volumes compared to traditional electrochemical cells (81), translate and amplify biological signals into electrical responses where direct or shuttling EET reduces the conductance of a conducting polymer channel (81). Conductance was measured under constant bias voltages (V_DS_ = −0.05V, V_GS_ = 0.2V). Both *C. sakazakii* and *V. fessus* exhibited a marked drop in conductance over 24 h; however, only *V. fessus* outperformed *E. coli* in these devices (Fig. 5a and b). To obtain a precise measurement of the electroactive activities, the transfer curves were plotted against the Ag/AgCl reference electrode (RE) (Fig. S8). A more positive effective gate voltage (V_G_^EFF^) or a more negative measured source electrode potential (V_S_) indicates a reduction after incubation with the bacteria (81). Together, these results suggest that these bacteria are capable of reducing soluble Fe(III) to Fe(II) and that, under our conditions, *V. fessus* exhibits similar EET-levels to *S. oneidensis* in reducing insoluble electron acceptors at the gate and channel of the OECT devices. In each evaluation, the same trends were observed with both *V. fessus* and *C. sakazakii* representing relatively weak electrogenic behavior.

## DISCUSSION

Common methods for characterizing phenotypic differences within a complex population, such as FACS (82), are challenging to apply to extracellular processes like EET. Addressing this challenge, microdroplet emulsions encapsulate individual cells into spatially isolated pico- to nano-liter droplets, allowing for smaller reaction sizes and higher sample numbers compared to 96-well (83) or 384-well plates (80, 84). To apply microdroplet emulsions toward the study of EET, we first overcame the dual challenges of developing a fluorescent output linked to EET activity and adapting emulsion preparation to oxygen-limited conditions. To address the first challenge, we leveraged our previous work showing that EET from *S. oneidensis* could control Cu(I)-catalyzed cycloaddition via *in situ* reduction of Cu(II) to Cu(I). In the presence of an appropriate substrate (CalFluor488 [55]), this reaction results in a quenched-to-fluorescent product. However, this reaction, as well as the expression of many EET genes, requires anaerobic conditions. Accordingly, we observed virtually no fluorescent signal when droplets were prepared under aerobic conditions. To circumvent this problem, we developed an oxygen-limited benchtop method by degassing the starting solutions and plumbing the microfluidic system under positive pressure (Fig. 1; Fig. S3; Fig. 2a through c). The resulting closed-loop system maintained anaerobic conditions through sample preparation, sorting, and collection while allowing for the growth of facultative and obligate anaerobes. Together, the combination of anaerobic conditions with an EET-driven fluorescent output established the minimum requirements for a microdroplet-based screen of EET activity. While we applied this system to the study of EET, we note that the oxygen-limited protocols reported here could also be used for adapting microdroplet emulsions for the characterization of gut, soil, and marine bacteria.

We initially validated fluorescent-based sorting of the microdroplet emulsion by examining mixed populations of *S. oneidensis* MR-1 and EET-deficient knockouts (∆*mtrC*∆*mtrF*∆*omcA* and ∆Mtr-pathway). We measured an approximately threefold and fourfold enrichment of the wild type in the sorted population (respectively), validating our ability to sort for EET activity in droplets. The modest enrichment after a single sort is likely due to spurious Cu(II) reduction from other EET proteins (e.g., MtrA and MtrD (19, 23, 70, 80, 85)) and can be further improved by iterative rounds of sorting. However, our single enrichment for *S. oneidensis* MR-1 indicates that despite this background reduction, the microdroplet system can capture electrogens from a mixed population. To improve enrichment in the future, stricter sorts involving multiple sequential FADS or multi-dimensional sorting could be used to isolate a more selective subset of bacterial species.

Microfluidic systems are ideal for avoiding microbe-microbe competition for resources, which can mute studies of their phenotypic behavior (51, 53, 82). We demonstrated this by performing microdroplet encapsulation on a sample of lake water and comparing the sorted population to one grown using bulk enrichment on Fe(III) (Fig. 4a). Encapsulation alone (prior to FADS) enriched notably for *Pseudomonas* (76.1% of the encapsulated population). These results are not surprising given that certain *Pseudomonas* species are known to secrete Cu-binding small molecules and the assay exposes the bacteria to a relatively high concentration of copper (86–88). Despite the large percentage of *Pseudomonads* in the droplet population, no *Pseudomonads* were detected in any of the fluorescent-sorted populations. Regardless of the growth-based advantage observed with *Pseudomonads* during droplet creation, our results highlight the utility of the microfluidic system for separating growth from function.

We identified several interesting bacteria from droplet-based sorting of our environmental sample. The ratios of bacteria that were strict anaerobes to aero-tolerant organisms stayed consistent between the starting population and the enriched population (Fig. 4d through f). However, there was a relative decrease in the number of strict anaerobes in the bulk enrichment. We measured a small relative percentage of *Geobacter* and *Shewanella* species in the initial isolate, but neither was enriched in the bulk enrichment or FADS. This could indicate that these species were not viable in the initial isolate, did not survive droplet encapsulation, or were not competitive in the bulk enrichment. Several bacteria were identified by both droplet and bulk enrichment that have not previously been reported as EET-capable. For example, *Acinetobacter lwoffii*, which was originally isolated from an arsenic-polluted environment, was enriched in both droplet and bulk (89). We also measured a large relative increase in our microdroplet screen for *Granulicatella adiacens*, which has been reported in several cases of infection surrounding metallic hip replacements (90). Interestingly, this species was present in significantly lower quantities in the bulk enrichment population, possibly due to its notorious difficulties in culturing under laboratory conditions (91). Furthermore, *Exiguobacterium indicum*, which has been shown to bio-reduce hexavalent chromium (92), was isolated by both FADS and bulk enrichment. *E. indicum* is also referenced as having the ability to reduce azo dyes (93), an ability shared by *Shewanella* species and sometimes attributed to EET (94, 95). In addition, known EET-capable organisms such as *Ochrobactrum* species were enriched only in the microdroplet system where we observed a greater than 1,000-fold enrichment (18).

At the population level, we used FeGenie (45), and example genomes to characterize differences between the droplet and bulk enrichments. Example genomes were gathered from the NIH National Center for Biotechnology Information (NCBI) and were limited only to representative genomes available, which most likely do not fully capture the information potential in the data set or the power FeGenie. Despite this limitation, we visualized significant differences between the relative genome percentages containing iron-reduction-related proteins or pathways between the droplets and bulk enrichments. These pathways are primarily based on well-understood iron-reduction proteins from *Shewanella, Geobacter,* and *Lysteria*. Using FeGenie, we identified several purported electroactive species that were unique to the microdroplet system. Among these, we selected *C. sakazakii* and *V. fessus* for additional characterization. *C. sakazakii* is known to infect infant formula and has previously been the focus of study regarding its iron acquisition systems and the ability for ferric iron to disrupt biofilm formation (77, 78). However, its ability to generate current or perform EET has not been reported. Similarly, to the best of our knowledge, there has not been a report of *V. fessus* displaying any iron-specific, heavy metal tolerance, or current generation. In MFCs, *V. fessus* and *C. sakazakii* both outperformed *E. coli* and underperformed *S. oneidensis* indicating weak electrogenic behavior. Both bacteria generated Fe(II) from soluble Fe(III), although again *C. sakazakii* only marginally outperformed the negative control of *E. coli*. Neither bacteria, under our anaerobic culturing conditions, was able to utilize Fe(III) as its sole terminal electron acceptor. Notably, we were unable to culture *V. fessus* without the presence of at least 0.1% sheep’s blood even when attempting to substitute with soluble Fe(III) citrate (Fig. S7). In monoculture in OECTs, *V. fessus* was able to reduce the current at a rate comparable to that of *S. oneidensis* while *C. sakazakii* only marginally outperformed the *E. coli* control. Further investigation into the genome of *V. fessus* revealed homology to gram-positive EET pathway proteins in a single operon: *dmkA*, *fmnB*, *pplA*, *ndh2*, *eetB*, and *dmkB*. Interestingly, although several other *Vagococcus* species have been implicated as EET-capable, *V. fessus* is not among them. Overall, these results highlight the ability of our droplet system to identify unique electroactive species and suggest electrogenic behavior from *V. fessus* and *C. sakazakii*.

We developed a high-throughput microdroplet emulsion screen for identifying electroactive bacteria from complex starting populations. The potentially different selective pressures between the droplet and bulk enrichment indicate it should serve to complement traditional EET screening such as growth on electrodes (32, 47, 96, 97), dialysis enrichments (63, 98, 99), growth on iron (35, 36, 100), and more. Relative to these techniques, the droplet system has a few limitations including the requirement for survival during emulsification, the relatively high concentration of Cu within the droplets, and the use of Cu(II) rather than Fe(III) for an extracellular electron acceptor. However, it has been previously seen that gram-positive EET-capable bacteria utilize EET to reduce Cu(II) for sensing by the CopRS system in *L. monocytogenes* (101, 102). Notably, current methods for identifying EET activity tend to rely on growth-related behavior, such as the ability to use Fe(III) as a sole terminal electron acceptor, or the ability to outcompete other bacteria within a bulk enrichment or in biofilm (7, 30, 103–105). There is an ongoing need to study both weak electrogens, and non-growth associated EET for their complex role in the environment, mineral-microbe interactions, and microbe-microbe interactions (26, 31, 46, 106). Furthermore, some bacteria exhibit EET only under certain conditions such as during pathogenesis (107) or with specific electron acceptors, which may be difficult to replicate under laboratory conditions (108). Overall, droplet-based screens may be an ideal method for establishing genotype-phenotype relationships for a wide variety of electroactive microbes.

## MATERIALS AND METHODS

CalFluor 488 (Click Chemistry Tools), alkyne-PEG4-acid (Click Chemistry Tools), copper(II) bromide (CuBr_2_, Sigma-Aldrich, 99%), 2-(4-((bis((1-(tert-butyl)−1 H-1,2,3-triazol-4-yl)methyl)amino)methyl)−1 H-1,2,3-triazol-1-yl)acetic acid (BTTAA, Click Chemistry Tools > 95%), sodium DL-lactate (NaC_3_H_5_O_3_, TCI, 60% in water), sodium fumarate (Na_2_C_4_H_2_O_4_,VWR, 98%), EPES buffer solution (C_8_H_18_N_2_O_4_S, VWR, 1 M in water, pH = 7.3), potassium phosphate dibasic (K_2_HPO_4_, Sigma-Aldrich), potassium phosphate monobasic (KH_2_PO_4_, Sigma-Aldrich), sodium chloride (NaCl, VWR), dimethyl sulfoxide (cell culture grade, Sigma-Aldrich), ammonium sulfate ((NH_4_)_2_SO_4_, Fisher Scientific), magnesium(II) sulfate heptahydrate (MgSO_4_·7H_2_O, VWR), trace mineral supplement (ATCC), casamino acids (VWR), Lysogeny Broth (BD), OptiPrep (Sigma-Aldrich), Pico-Gen 60 × 60 single aqueous (Sphere Fluidics), Pico-Wave (Sphere Fluidics), Pico-Break 1 (Sphere Fluidics), Pico-Mix (Sphere Fluidics), Pico-Surf (Sphere Fluidics), Medical Grade Polyethylene Micro Tubing/0.015″ ID x 0.043″ OD (±0.003″) =.38 mm ID x 1.09 mm OD (±.076 mm)/(100′ Roll) (Scientific Commodities), *Cronobacter sakazaki* (Farmer et al.) Iverson et al. (ATCC 29544), *Vagococcus fessus* Hoyles et al. (ATCC BAA-289), defibrillated sheep’s blood (Lampire), Tryptic Soy Broth (BD 211825), Nutrient Broth (BD cat 234000), Agar (BD), and disodium;4-[3-pyridin-2-yl-6-(4-sulfonatophenyl)−1,2,4-triazin-5-yl]benzenesulfonate hydrate (Ferrozine, VWR). All media components were autoclaved or sterilized using 0.2 µm PES filters.

### Oxygen-limited encapsulation of *S. oneidensis*

Overnight cultures were grown in a Coy Anaerobic Glovebox containing a humidified atmosphere at 3% hydrogen content and the balance nitrogen. The cultures were started by picking a single colony into argon-sparged LB broth supplemented with 20 mM sodium lactate (2.85 µL of 60% wt/wt sodium lactate per 1 mL culture). After overnight growth anaerobically at 30°C, cell cultures were diluted to an OD_600_ of 0.00006 into a solution of 40 mM lactate, 80 mM fumarate, and 20 wt% OptiPrep in LB broth. Inside the anaerobic chamber, 1 mL of the cell solution was loaded into the aqueous syringe (BD). The tubing was prepared by heat-sealing one end, and the other was loaded onto a needle, inside of the anaerobic glove box, the needle was attached to an empty syringe and pressure was pulled three times and held for 30 seconds each time and then vented after each round to remove any O_2_ present in the tubing. In addition, 10 mL of argon-sparged 2.5% 008-FluoroSurfactant in Pico-Wave was loaded into the oil syringe (SGE) and capped with a sealed sparged needle, and tubing. A collection syringe was prepared by pulling 6 mL of the Coy Anaerobic Glovebox atmosphere and capped with a sealed sparged needle, and tubing. A PicoDroplet Single Cell Encapsulation System (Sphere Fluidics) was used. All sealed syringes were removed from the anaerobic chamber. Each syringe was loaded under a 100 µL/h positive pressure before clipping the heat-sealed end and connecting to the 60 × 60 droplet maker (Pico-Gen) chip. To make the emulsion, the syringe pumps were increased to 1,000 µL/h (aqueous) and 1,200 µL/h (oil). The system was allowed to calibrate before cutting and plumbing the collection syringe (BD). After encapsulation, emulsions were incubated at 30°C 24 h to allow for growth within the droplets.

### Oxygen-limited Pico-injection of CuAAC components

A 5X Cu(I)-catalyzed Alkyne–Azide Cycloaddition solution (70 µM CalFluor 488, 2 mM Alkyne-Peg4-acid, 2 mM Cu:BTTAA (1:6)) was prepared from a 3.2 mM stock of CalFluor 488 in DMSO, 8 mM stock of CuBr_2_ in water, 48 mM stock of BTTAA in water, and a 4 mM stock of Alkyne-Peg_4_-Acid in water in a Coy Anaerobic Glovebox containing a humidified atmosphere at 3% hydrogen content and the balance nitrogen. Into a 1 mL syringe, 200 µL of the solution was loaded into the aqueous syringe (BD) and capped with a sealed sparged needle, and tubing. In addition, 10 mL of argon-sparged Pico-Wave was loaded into the oil syringe (SGE) and capped with a sealed sparged needle, and tubing. A collection syringe (12 mL Manufactuer) was prepared by pulling 6 mL of the Coy Anaerobic Glovebox atmosphere, adding in 500 µL of 008-Fluorosurfactant (5%) in Pico-Wave, and capped with a sealed sparged needle, and tubing. The droplets were then transferred to a 1 mL syringe (BD) and capped with a sealed sparged needle, and tubing. All sealed syringes were removed from the anaerobic chamber, and loaded onto the syringe pumps set at 15 µL/h (aqueous), 100 µL/h (droplets), and 1,200 µL/h (oil) before cutting off the sealed end and plumbing into a Pico-Mix chip. The system was allowed to calibrate before cutting and plumbing the collection syringe (PGE). After pico-injection, emulsions were incubated at 30°C 24 h to allow for growth within the droplets.

### Fluorescent sorting for heterogeneity

For droplet sorting, a Single-Cell Assay and Isolation platform (Sphere Fluidics) with a 488 nm laser 244 and 525/50 emission filter (GFP) was used. PMT setup of the system was set to a gain of 0.8 or 1 such that an appropriate spread was seen without maxing out the detector for both PMT 1 (GFP channel/bandpass 525/50) and PMT 2 (large bandpass 650/150). The peak detection minimum was set at 0.07 and the maximum at 100. The minimum width was set to 0.18 and the maximum set to 100. Sorting gates were determined based on population distribution and manually drawn above 1 V(MR-1), 2.1V, 3.5 V (Lake water), 4.25 V (Lake water enrichment round 2). The top 3,000 droplets were then collected using an applied voltage of 0.3V. After sorting, the emulsion was broken with 100 µL Pico-Break (Sphere Fluidics) and was extracted for sequencing or cultured for further analysis. Culturing occurred in LB broth supplemented with 20 mM lactate at 30°C overnight. Each sample was split, freezing 500 µL as a cryogenic stock, and diluting 10 µL into the anaerobic chamber to start the creation of the next generation of sorts.

### Lake water enrichment

Samples were collected at Lat/Long: 30.273670,–97.770392. To obtain a natural, mixed community of bacteria, sediment samples were collected from Town Lake in Austin, TX on the morning of May 8, 2023. The ambient temperature at the time of sampling was 75°F (23.9°C). This lake has a near-neutral average pH of 7.5, and the sediment has an iron concentration of 5.2 g Fe/kg sediment. Triplicate samples were collected in glass jars at a water depth of 1–2′. Sediments from the first 4″ of sediment were scooped into jars and sealed underwater. Samples were placed on ice and brought back to the laboratory where excess water was removed in an anaerobic chamber, and samples were stored in the dark and at −20°C until use in an effort to minimize microbial change.

Environmental samples were enriched in an anaerobic bulk system microcosm similar to those previously described (49). Briefly, 10 mL of sediment-containing lake water containing a sediment-associated mixed microbial community was inoculated into a 250 mL anaerobic bulk systems filled with filter-sterilized lake water. The cells were provided with lactate at 0.1 mM as a carbon source, and iron-containing sediments were isolated in 3.5 kDa dialysis tubing. The cells were allowed to grow for 5 days and redox potential, pH, and aqueous Fe(II) concentrations were measured daily.

### 16S sample extraction and sequencing

DNA samples from both pre- and post-enriched samples via the oxygen-limited droplet protocol and bulk sedimentation enrichments were purified via the AllPrep DNA/RNA kit from Qiagen per the manufacturer’s instructions. Samples were sent to Mr. DNA for microbial taxonomic analysis. Mr. DNA utilizes 515 F-806R (V4 region) primers, and classification is determined using QIIME (109). Taxonomic classification from Mr. DNA was utilized at the genus and species level for all subsequent analyses.

Species richness for each sample was estimated using a rarefaction curve based on species-level counts. The rarefaction curve data were generated using the “rarecurve” function in the vegan (v. 2.6.4) (110) library with default settings. Taxonomic proportions were calculated by dividing counts from each individual taxa by the total number of read counts for that sample. We estimated a species to show prospective “enrichment” for downstream testing within a sample based on the following criteria: if a certain taxon was above 0.015% of the initial population, and also showed a higher proportion in the compared population of interest. We estimated this initial population detection cutoff based on the initial library size and the consistency of the number of enrichments for a specified sample. Taxa of interest were also selected if they showed notable non-zero detection within samples of interest while showing no observance in reference samples. Species enrichments were used to obtain a list of genomes to later be used for FeGenie. Analyses were performed using custom scripts in Python and R. Taxonomic barplots and rarefaction plots were generated using ggplot2 (v. 3.5.0) (111). The EET species database was generated via Genomic sequences from these taxa were collected for any full genome or 16S (full or V4) sequences available on NCBI from the following citations (17, 18, 27, 32, 46, 65–68, 106, 112). Plots were generated in R.

### FeGenie

Example genomes were obtained using NCBI and chosen based on the longest whole-genome sequencing available for a given genome. If the species level was not specified, the most appropriate example genome was chosen from the genus. Each genome was exported and FeGenie was run on Python3 and the code was available for download on GitHub. Results were then processed to normalize the number of genes per genome analyzed and represented as percentages. These graphs were created using Python3 and the code is available in the SI. Relative fold-enrichment was calculated by normalizing the enriched population by the starting population.

### OECT device operation and electrochemistry

Two different types of electrolytes were used according to the bacterial strains. Medium 3 was used with *Cronobacter sakazakii*, *S. oneidensis* MR-1*,* and *E. coli*. A mixture of medium 260 and Luria-Bertani (LB) broth at 1:50 was used with *Vagococcus fessus*, *S. oneidensis*, and *E. coli*. All electrolytes were purged with argon bubbling for at least 15 min before use. Before each experiment, the OECT slides and PDMS layer were autoclaved separately and subsequently assembled in the biosafety cabinet. The fabrication of devices is outlined further in the Supplementary Information. To ensure an oxygen-free environment, the OECT experiments were carried out inside the nitrogen-filled glovebox. Electrochemical measurements were conducted with the multichannel potentiostat (MultiPalmSens4, PalmSens BV). Prior to inoculation, OECTs were equilibrated in the glovebox with abiotic electrolytes for 3 days. For the inoculation process of the OECTs, all cells were grown anaerobically overnight in their respective media. Grown cell culture cell density (OD_600_) was measured, and the cells were concentrated and resuspended to the intended OD_600_ at 0.1 with the respective purged media, forming the inoculum culture. Subsequently, the inoculum cultures were used to inoculate OECTs at a 1:9 ratio of cell culture to the OECT electrolyte, achieving an intended inoculation OD_600_ at 0.01. During the OECT experiment, the gate voltage VGS and drain voltage VDS were constantly biased at 0.2 V and −0.05 V, respectively. Transfer curve measurements were conducted 24 h after inoculation, with the V_DS_ kept at −0.05 V while the V_GS_ scanned from −0.1 V to 0.6 V with a scan rate of 20 mV/s. The open circuit potentials (OCP) of the source and gate electrode potentials were measured against the Ag/AgCl pellet reference electrodes (RE) (550010, A-M Systems). The Ag/AgCl electrodes were directly inserted into the OECT chamber without using any salt bridges.

### MFC operation

Two-chamber H-cell MFCs were comprised of two glass cylinders serving as the anode and cathode chambers, connected via side-arm tubes with an inner diameter of 30 mm. The chambers were separated by a proton exchange membrane (PEM) (Nafion 117), which was secured between the side arms using rubber O-rings on both sides to ensure a tight seal. The operational volume of each chamber was 200 mL. Graphite blocks (dimensions: 12 × 45 × 57 mm³) were employed as the anode electrodes, while carbon-pellet-filled stainless steel pouches were used as the cathode electrodes. Electrical loads were connected via resistors (0.25 W, ±1% tolerance) with customized connectors. For the catholyte, filter-sterilized 0.1 M potassium ferricyanide was used. The anolyte consisted of the respective sterile growth media (LB, media 3, or media 260) depending on the strains to be tested. Before each experiment, the MFC chambers, electrodes, and O-rings were thoroughly washed with soapy water, rinsed with ultrapure water, and sterilized by autoclaving. The PEMs were pretreated by immersion in 0.1 M H₂SO₄ for 1 h to enhance proton conductivity and were subsequently stored in sterile ultrapure water until use. After sterilization, the MFCs were assembled without filling the electrolytes and transferred into a nitrogen-filled glovebox to maintain an oxygen-free environment. Experiments were conducted in a glovebox at room temperature. The pre-assembled MFCs were brought into the glovebox and filled with anolyte and catholyte. Pre-cultured bacterial cells (5 mL) grown aerobically were harvested by centrifugation (6,000 × *g*, 10 min), and the resulting cell pellets were transferred into the glovebox for inoculation into the anode chambers. External resistor loads of 10 kΩ were connected throughout the experiment except during polarization curve measurements. A multichannel potentiostat (MultiPalmSens4, PalmSens BV) was connected to the external resistors to monitor the load voltages, and the corresponding load currents were calculated using Ohm’s law. The anolyte was replaced to maintain optimal conditions when the load current showed a decline. During the first anolyte replacement, half the volume (100 mL) was replaced, in the subsequent replacements the entire anolyte (200 mL) was replaced. Polarization curves were measured during the plateau phase of the load current.

### Ferrozine assay

Aerobic cultures of *S. oneidensis, E. coli*, *C. sakazakii*, and *V. fessus* were created in each bacterium’s preferred rich media (LB, LB, Media 3, and Media 260, respectively). The next day, cultures were diluted 1/100 into anaerobic media and allowed to grow overnight. Each bacterium was washed via centrifugation at 6,000 rcf and decanted before the supernatant was exchanged for the reaction media (Media 3 for *C. sakazakii* and Media 260:LB 1:50 for *V. fessus*). Cultures of *S. oneidensis* and *E. coli* were also washed and reconstituted in each media to be run concurrently. Ferrozine was dissolved into degassed, anaerobic growth media such that the final concentration of the assay could be run at 1 mg/mL. 6.57 µL of 190 mM Fe(III)citrate was added in addition to 10 µL of cells directly from aerobic overnights into a 250 µL reaction in a clear-bottom U-shaped Grenier 96-well plate (final Fe(III) concentration of 2 mM). A calibration curve of Fe(II) sulfate stocks in sterile water was inoculated into control wells for each media containing ferrozine from 0 µM-96 µM. Each media had a cell-free control inoculated with Fe(III) but no cells. The reduction of Fe(II) was measured using the calibration curve minus the background reduction via the media blank. The complete 96-well plate was sealed anaerobically and placed into an incubated plate reader. Readings were taken every 3 min at 562 nm. The mixed cultures of ∆*mtrC*∆*mtrF*∆*omcA* and wild-type MR-1 were grown overnight in *Shewanella* Basal Media (SBM) supplemented with 0.05% casamino acids and Wolfe’s Mineral Solution. The cells were used without washing and subjected to the same treatment in SBM with casamino acids and mineral solution.

### Growth on Fe(III)

To a 96-well plate, 6.57 µL of 190 mM Fe(III)citrate was added in addition to 10 µL of cells directly from aerobic overnights into a 250 µL reaction in a clear-bottom U shaped Grenier 96-well plate in the anaerobic growth media (Media 3 for *C. sakazakii,* and 1:50 Media 260:LB for *V. fessus*). Fe(III)-free wells were made with the addition of water in the place of Fe(III). Media controls were included for blanking the readings. The complete 96-well plate was sealed anaerobically and placed into an incubated plate reader. Readings were taken every 3 min at 600 nm.

## Data Availability

Experimental data supporting the findings of this study are available through the Texas Data Repository at https://doi.org/10.18738/T8/VI9BJM.

## SUPPLEMENTAL MATERIAL

Supplemental material aem.02465-24-s0001.pdf Supplemental methods, Table S1, Fig. S1 to S9, and code availability.

Video S1 aem.02465-24-s0002.mp4 Video of bacteria moving within the droplets immediately prior to sort of a ∆Mtr-pathway:MR-1 mixture sort.

ASM does not own the copyrights to Supplemental Material that may be linked to, or accessed through, an article. The authors have granted ASM a non-exclusive, world-wide license to publish the Supplemental Material files. Please contact the corresponding author directly for reuse.

## References

[1] Shi L, Dong H, Reguera G, Beyenal H, Lu A, Liu J, Yu H-Q, Fredrickson JK. 2016. Extracellular electron transfer mechanisms between microorganisms and minerals. Nat Rev Microbiol 14:651–662. doi:10.1038/nrmicro.2016.93 27573579

[2] Choi S. 2022. Electrogenic bacteria promise new opportunities for powering, sensing, and synthesizing. Small 18:e2107902. doi:10.1002/smll.202107902 35119203

[3] Itoh K, Kitade Y, Nakanishi M, Yatome C. 2002. Decolorization of methyl red by a mixed culture of Bacillus sp. and Pseudomonas stutzeri. J Environ Sci Health - Part Toxic Hazard Subst Environ Eng 37:415–421. doi:10.1081/ESE-120002838 11929077

[4] Morrison JM, John GH. 2016. Growth and physiology of Clostridium perfringens wild-type and ΔazoC knockout: an azo dye exposure study. Microbiol (Reading) 162:330–338. doi:10.1099/mic.0.000212 26566621

[5] Yoo ES, Libra J, Adrian L. 2001. Mechanism of decolorization of azo dyes in anaerobic mixed culture. J Environ Eng 127:844–849. doi:10.1061/(ASCE)0733-9372(2001)127:9(844)

[6] Gingell R, Walker R. 1971. Mechanisms of azo reduction by Streptococcus faecalis. II. The role of soluble flavins. Xenobiot 1:231–239. doi:10.3109/00498257109033172 4341449

[7] Khan MT, Duncan SH, Stams AJM, van Dijl JM, Flint HJ, Harmsen HJM. 2012. The gut anaerobe Faecalibacterium prausnitzii uses an extracellular electron shuttle to grow at oxic-anoxic interphases. ISME J 6:1578–1585. doi:10.1038/ismej.2012.5 22357539 PMC3400418

[8] Naradasu D, Miran W, Sakamoto M, Okamoto A. 2018. Isolation and characterization of human gut bacteria capable of extracellular electron transport by electrochemical techniques. Front Microbiol 9:3267. doi:10.3389/fmicb.2018.03267 30697198 PMC6340925

[9] Winter SE, Thiennimitr P, Winter MG, Butler BP, Huseby DL, Crawford RW, Russell JM, Bevins CL, Adams LG, Tsolis RM, Roth JR, Bäumler AJ. 2010. Gut inflammation provides a respiratory electron acceptor for Salmonella. Nature New Biol 467:426–429. doi:10.1038/nature09415 PMC2946174 20864996

[10] Tahernia M, Plotkin-Kaye E, Mohammadifar M, Gao Y, Oefelein MR, Cook LC, Choi S. 2020. Characterization of electrogenic gut bacteria. ACS Omega 5:29439–29446. doi:10.1021/acsomega.0c04362 33225175 PMC7676329

[11] Garbini GL, Barra Caracciolo A, Grenni P. 2023. Electroactive bacteria in natural ecosystems and their applications in microbial fuel cells for bioremediation: a review. Microorganisms 11:1255. doi:10.3390/microorganisms11051255 37317229 PMC10263229

[12] Liu J, Fan L, Yin W, Zhang S, Su X, Lin H, Yu H, Jiang Z, Sun F. 2023. Anaerobic biodegradation of azo dye reactive black 5 by a novel strain Shewanella sp. SR1: pathway and mechanisms. J Environ Manage 347:119073. doi:10.1016/j.jenvman.2023.119073 37776795

[13] Hu Y, Yang Y, Katz E, Song H. 2015. Programming the quorum sensing-based AND gate in Shewanella oneidensis for logic gated-microbial fuel cells. Chem Commun 51:4184–4187. doi:10.1039/C5CC00026B 25673159

[14] Newton GJ, Mori S, Nakamura R, Hashimoto K, Watanabe K. 2009. Analyses of current-generating mechanisms of Shewanella loihica PV-4 and Shewanella oneidensis MR-1 in microbial fuel cells. Appl Environ Microbiol 75:7674–7681. doi:10.1128/AEM.01142-09 19837834 PMC2794086

[15] Reguera G, Nevin KP, Nicoll JS, Covalla SF, Woodard TL, Lovley DR. 2006. Biofilm and nanowire production leads to increased current in Geobacter sulfurreducens fuel cells. Appl Environ Microbiol 72:7345–7348. doi:10.1128/AEM.01444-06 16936064 PMC1636155

[16] Uría N, Muñoz Berbel X, Sánchez O, Muñoz FX, Mas J. 2011. Transient storage of electrical charge in biofilms of Shewanella oneidensis MR-1 growing in a microbial fuel cell. Environ Sci Technol 45:10250–10256. doi:10.1021/es2025214 21981730

[17] Yilmazel YD, Zhu X, Kim K-Y, Holmes DE, Logan BE. 2018. Electrical current generation in microbial electrolysis cells by hyperthermophilic archaea Ferroglobus placidus and Geoglobus ahangari. Bioelectrochemistry 119:142–149. doi:10.1016/j.bioelechem.2017.09.012 28992595

[18] Zuo Y, Xing D, Regan JM, Logan BE. 2008. Isolation of the exoelectrogenic bacterium Ochrobactrum anthropi YZ-1 by using a U-tube microbial fuel cell. Appl Environ Microbiol 74:3130–3137. doi:10.1128/AEM.02732-07 18359834 PMC2394939

[19] Dundas CM, Walker DJF, Keitz BK. 2020. Tuning extracellular electron transfer by Shewanella oneidensis using transcriptional logic gates. ACS Synth Biol 9:2301–2315. doi:10.1021/acssynbio.9b00517 32786362 PMC7816516

[20] Fan G, Dundas CM, Graham AJ, Lynd NA, Keitz BK. 2018. Shewanella oneidensis as a living electrode for controlled radical polymerization. Proc Natl Acad Sci U S A 115:4559–4564. doi:10.1073/pnas.1800869115 29666254 PMC5939106

[21] Fan G, Graham AJ, Kolli J, Lynd NA, Keitz BK. 2020. Aerobic radical polymerization mediated by microbial metabolism. Nat Chem 12:638–646. doi:10.1038/s41557-020-0460-1 32424254 PMC7321916

[22] Graham AJ, Dundas CM, Partipilo G, Miniel Mahfoud IE, FitzSimons T, Rinehart R, Chiu D, Tyndall AE, Rosales AM, Keitz BK. 2021. Transcriptional regulation of synthetic polymer networks. Synth Biol. doi:10.1101/2021.10.17.464678

[23] Partipilo G, Graham AJ, Belardi B, Keitz BK. 2022. Extracellular electron transfer enables cellular control of Cu(I)-catalyzed alkyne-azide cycloaddition. ACS Cent Sci 8:246–257. doi:10.1021/acscentsci.1c01208 35233456 PMC8875427

[24] Brewster RC, Suitor JT, Bennett AW, Wallace S. 2019. Transition metal‐free reduction of activated alkenes using a living microorganism. Angew Chem Int Ed 58:12409–12414. doi:10.1002/anie.201903973 31286626

[25] Sadler JC, Dennis JA, Johnson NW, Wallace S. 2021. Interfacing non-enzymatic catalysis with living microorganisms. RSC Chem Biol 2:1073–1083. doi:10.1039/d1cb00072a 34458824 PMC8341791

[26] Doyle LE, Marsili E. 2018. Weak electricigens: a new avenue for bioelectrochemical research. Bioresour Technol 258:354–364. doi:10.1016/j.biortech.2018.02.073 29519634

[27] Koch C, Harnisch F. 2016. Is there a specific ecological niche for electroactive microorganisms? ChemElectroChem 3:1282–1295. doi:10.1002/celc.201600079

[28] Zhou AY, Baruch M, Ajo-Franklin CM, Maharbiz MM. 2017. A portable bioelectronic sensing system (BESSY) for environmental deployment incorporating differential microbial sensing in miniaturized reactors. PLOS ONE 12:e0184994. doi:10.1371/journal.pone.0184994 28915277 PMC5600388

[29] Reniere MLR. 2018. Reduce, induce, thrive: bacterial redox sensing during pathogenesis. J Bacteriol 200:1–12. doi:10.1128/JB.00128-18 PMC6088161 29891640

[30] Zhang S, Miran W, Naradasu D, Guo S, Okamoto A. 2020. A human pathogen Capnocytophaga ochracea exhibits current producing capability. Electrochem 88:224–229. doi:10.5796/electrochemistry.20-00021

[31] Aiyer K, Doyle LE. 2023. Extracellular electron transfer of weak electricigens in the presence of a competing electron acceptor. J Electrochem Soc 170:055501. doi:10.1149/1945-7111/accf3e

[32] Babu Arulmani SR, Jayaraj V, Jebakumar SR. 2016. Long-term electricity production from soil electrogenic bacteria and high-content screening of biofilm formation on the electrodes. J Soils Sediments 16:831–841. doi:10.1007/s11368-015-1287-z

[33] Badalamenti JP, Summers ZM, Chan CH, Gralnick JA, Bond DR. 2016. Isolation and genomic characterization of “Desulfuromonas soudanensis WTL”, a metal- and electrode-respiring bacterium from anoxic deep subsurface brine. Front Microbiol 7:913. doi:10.3389/fmicb.2016.00913 27445996 PMC4914508

[34] Brutinel ED, Gralnick JA. 2012. Shuttling happens: soluble flavin mediators of extracellular electron transfer in Shewanella. Appl Microbiol Biotechnol 93:41–48. doi:10.1007/s00253-011-3653-0 22072194

[35] Chabert N, Amin Ali O, Achouak W. 2015. All ecosystems potentially host electrogenic bacteria. Bioelectrochemistry 106:88–96. doi:10.1016/j.bioelechem.2015.07.004 26298511

[36] Fredrickson JK, Zachara JM. 2008. Electron transfer at the microbe-mineral interface: a grand challenge in biogeochemistry. Geobiology 6:245–253. doi:10.1111/j.1472-4669.2008.00146.x 18498527

[37] Kiely PD, Regan JM, Logan BE. 2011. The electric picnic: synergistic requirements for exoelectrogenic microbial communities. Curr Opin Biotechnol 22:378–385. doi:10.1016/j.copbio.2011.03.003 21441020

[38] Logan BE. 2009. Exoelectrogenic bacteria that power microbial fuel cells. Nat Rev Microbiol 7:375–381. doi:10.1038/nrmicro2113 19330018

[39] Sacco NJ, Bonetto MC, Cortón EI, Sp D. 2017. Isolation and characterization of a novel electrogenic bacterium, Dietzia sp. RNV-4. PLOS ONE 12:e0169955. doi:10.1371/journal.pone.0169955 28192491 PMC5305051

[40] Yadav S, Patil SA. 2020. Microbial electroactive biofilms dominated by Geoalkalibacter spp. from a highly saline-alkaline environment. NPJ Biofilms Microbiomes 6:38. doi:10.1038/s41522-020-00147-7 33051461 PMC7555509

[41] Edel M, Sturm G, Sturm-Richter K, Wagner M, Ducassou JN, Couté Y, Horn H, Gescher J. 2021. Extracellular riboflavin induces anaerobic biofilm formation in Shewanella oneidensis. Biotechnol Biofuels 14:130. doi:10.1186/s13068-021-01981-3 34082787 PMC8176591

[42] Kees ED, Levar CE, Miller SP, Bond DR, Gralnick JA, Dean AM. 2021. Survival of the first rather than the fittest in a Shewanella electrode biofilm. Commun Biol 4:536. doi:10.1038/s42003-021-02040-1 33958697 PMC8102560

[43] Snider RM, Strycharz-Glaven SM, Tsoi SD, Erickson JS, Tender LM. 2012. Long-range electron transport in Geobacter sulfurreducens biofilms is redox gradient-driven. Proc Natl Acad Sci U S A 109:15467–15472. doi:10.1073/pnas.1209829109 22955881 PMC3458377

[44] Engel C, Schattenberg F, Dohnt K, Schröder U, Müller S, Krull R. 2019. Long-term behavior of defined mixed cultures of Geobacter sulfurreducens and Shewanella oneidensis in bioelectrochemical systems. Front Bioeng Biotechnol 7:60. doi:10.3389/fbioe.2019.00060 30972336 PMC6445848

[45] Garber AI, Nealson KH, Okamoto A, McAllister SM, Chan CS, Barco RA, Merino N. 2020. FeGenie: a comprehensive tool for the identification of iron genes and iron gene neighborhoods in genome and metagenome assemblies. Front Microbiol 11:37. doi:10.3389/fmicb.2020.00037 32082281 PMC7005843

[46] Light SH, Su L, Rivera-Lugo R, Cornejo JA, Louie A, Iavarone AT, Ajo-Franklin CM, Portnoy DA. 2018. A flavin-based extracellular electron transfer mechanism in diverse Gram-positive bacteria. Nature New Biol 562:140–144. doi:10.1038/s41586-018-0498-z PMC6221200 30209391

[47] Holmes DE, Zhou J, Smith JA, Wang C, Liu X, Lovley DR. 2022. Different routes for direct interspecies electron transfer with diverse electron-accepting partners. Microbiology. doi:10.1101/2022.04.27.489562

[48] Wei L, Han H, Shen J. 2012. Effects of cathodic electron acceptors and potassium ferricyanide concentrations on the performance of microbial fuel cell. Int J Hydrogen Energy 37:12980–12986. doi:10.1016/j.ijhydene.2012.05.068

[49] Igarashi K, Kato S. 2021. Reductive transformation of Fe(III) (oxyhydr)oxides by mesophilic homoacetogens in the genus Sporomusa. Front Microbiol 12:600808. doi:10.3389/fmicb.2021.600808 33633701 PMC7901989

[50] Bowman EK, Wagner JM, Yuan S-F, Deaner M, Palmer CM, D’Oelsnitz S, Cordova L, Li X, Craig FF, Alper HS. 2021. Sorting for secreted molecule production using a biosensor-in-microdroplet approach. Proc Natl Acad Sci U S A 118:118. doi:10.1073/pnas.2106818118 PMC8433520 34475218

[51] Matuła K, Rivello F, Huck WTS. 2020. Droplet microfluidics: single‐cell analysis using droplet microfluidics (Adv. Biosys. 1/2020). Adv Biosys 4:2070012. doi:10.1002/adbi.202070012 32293129

[52] Najah M, Calbrix R, Mahendra-Wijaya IP, Beneyton T, Griffiths AD, Drevelle A. 2014. Droplet-based microfluidics platform for ultra-high-throughput bioprospecting of cellulolytic microorganisms. Chem Biol 21:1722–1732. doi:10.1016/j.chembiol.2014.10.020 25525991

[53] Liu Y, Fan Z, Qiao L, Liu B. 2022. Advances in microfluidic strategies for single-cell research. TrAC 157:116822. doi:10.1016/j.trac.2022.116822

[54] Kleyer H, Tecon R, Or D. 2017. Resolving species level changes in a representative soil bacterial community using microfluidic quantitative PCR. Front Microbiol 8:2017. doi:10.3389/fmicb.2017.02017 29118739 PMC5661172

[55] Shieh P, Dien VT, Beahm BJ, Castellano JM, Wyss-Coray T, Bertozzi CR. 2015. CalFluors: a universal motif for fluorogenic azide probes across the visible spectrum. J Am Chem Soc 137:7145–7151. doi:10.1021/jacs.5b02383 25902190 PMC4487548

[56] Wagner JM, Liu L, Yuan S-F, Venkataraman MV, Abate AR, Alper HS. 2018. A comparative analysis of single cell and droplet-based FACS for improving production phenotypes: riboflavin overproduction in Yarrowia lipolytic. Metab Eng 47:346–356. doi:10.1016/j.ymben.2018.04.015 29698778

[57] Grigorov E, Kirov B, Marinov MB, Galabov V. 2021. Review of microfluidic methods for cellular lysis. Micromachines (Basel) 12:498. doi:10.3390/mi12050498 33925101 PMC8145176

[58] Nan L, Jiang Z, Wei X. 2014. Emerging microfluidic devices for cell lysis: a review. Lab Chip 14:1060–1073. doi:10.1039/c3lc51133b 24480982

[59] Bowman EK, Nguyen Hoang PT, Gordillo Sierra AR, Vieira Nogueira KM, Alper HS. 2023. Temporal sorting of microdroplets can identify productivity differences of itaconic acid from libraries of Yarrowia lipolytica. Lab Chip 23:2249–2256. doi:10.1039/d3lc00020f 37013836

[60] Beneyton T, Thomas S, Griffiths AD, Nicaud J-M, Drevelle A, Rossignol T. 2017. Droplet-based microfluidic high-throughput screening of heterologous enzymes secreted by the yeast Yarrowia lipolytica. Microb Cell Fact 16:18. doi:10.1186/s12934-017-0629-5 28143479 PMC5282883

[61] Stucki A, Vallapurackal J, Ward TR, Dittrich PS. 2021. Droplet microfluidics and directed evolution of enzymes: an intertwined journey. Angew Chem Int Ed Engl 60:24368–24387. doi:10.1002/anie.202016154 33539653 PMC8596820

[62] Agresti JJ, Antipov E, Abate AR, Ahn K, Rowat AC, Baret J-C, Marquez M, Klibanov AM, Griffiths AD, Weitz DA. 2010. Ultrahigh-throughput screening in drop-based microfluidics for directed evolution. Proc Natl Acad Sci USA 107:4004–4009. doi:10.1073/pnas.0910781107 20142500 PMC2840095

[63] Arnold RG, DiChristina TJ, Hoffmann MR. 1986. Inhibitor studies of dissimilative Fe(III) reduction by Pseudomonas sp. strain 200 (“Pseudomonas ferrireductans”). Appl Environ Microbiol 52:281–289. doi:10.1128/aem.52.2.281-289.1986 2428308 PMC203516

[64] Uria N, Ferrera I, Mas J. 2017. Electrochemical performance and microbial community profiles in microbial fuel cells in relation to electron transfer mechanisms. BMC Microbiol 17:208. doi:10.1186/s12866-017-1115-2 29047333 PMC5648455

[65] Lonergan DJ, Jenter HL, Coates JD, Phillips EJ, Schmidt TM, Lovley DR. 1996. Phylogenetic analysis of dissimilatory Fe(III)-reducing bacteria. J Bacteriol 178:2402–2408. doi:10.1128/jb.178.8.2402-2408.1996 8636045 PMC177952

[66] Sydow A, Krieg T, Mayer F, Schrader J, Holtmann D. 2014. Electroactive bacteria--molecular mechanisms and genetic tools. Appl Microbiol Biotechnol 98:8481–8495. doi:10.1007/s00253-014-6005-z 25139447

[67] He S, Barco RA, Emerson D, Roden EE. 2017. Comparative genomic analysis of neutrophilic iron(II) oxidizer genomes for candidate genes in extracellular electron transfer. Front Microbiol 8:1584. doi:10.3389/fmicb.2017.01584 28871245 PMC5566968

[68] Baker IR, Conley BE, Gralnick JA, Girguis PR. 2021. Evidence for horizontal and vertical transmission of Mtr- mediated extracellular electron transfer among the bacteria. MBio 13:e0290421. doi:10.1128/mbio.02904-21 35100867 PMC8805035

[69] Santos TC, Silva MA, Morgado L, Dantas JM, Salgueiro CA. 2015. Diving into the redox properties of geobacter sulfurreducens cytochromes: a model for extracellular electron transfer. Dalton Trans 44:9335–9344. doi:10.1039/c5dt00556f 25906375

[70] Pitts KE, Dobbin PS, Reyes-Ramirez F, Thomson AJ, Richardson DJ, Seward HE. 2003. Characterization of the Shewanella oneidensis MR-1 decaheme cytochrome MtrA. J Biol Chem 278:27758–27765. doi:10.1074/jbc.M302582200 12732647

[71] Castelle CJ, Roger M, Bauzan M, Brugna M, Lignon S, Nimtz M, Golyshina OV, Giudici-Orticoni M-T, Guiral M. 2015. The aerobic respiratory chain of the acidophilic archaeon Ferroplasma acidiphilum: a membrane-bound complex oxidizing ferrous iron. Biochim et Biophys Acta (BBA) - Bioenerg 1847:717–728. doi:10.1016/j.bbabio.2015.04.006 25896560

[72] Jiao Y, Newman DK. 2007. The Pio operon is essential for phototrophic Fe(II) oxidation in Rhodopseudomonas palustris TIE-1. J Bacteriol 189:1765–1773. doi:10.1128/JB.00776-06 17189359 PMC1855732

[73] Bewley KD, Firer-Sherwood MA, Mock J-Y, Ando N, Drennan CL, Elliott SJ. 2012. Mind the gap: diversity and reactivity relationships among multihaem cytochromes of the MtrA/DmsE family. Biochem Soc Trans 40:1268–1273. doi:10.1042/BST20120106 23176466 PMC5906043

[74] Beliaev AS, Thompson DK, Khare T, Lim H, Brandt CC, Li G, Murray AE, Heidelberg JF, Giometti CS, Yates J 3rd, Nealson KH, Tiedje JM, Zhoui J. 2002. Gene and protein expression profiles of Shewanella oneidensis during anaerobic growth with different electron acceptors. OMICS 6:39–60. doi:10.1089/15362310252780834 11881834

[75] Hartshorne RS, Jepson BN, Clarke TA, Field SJ, Fredrickson J, Zachara J, Shi L, Butt JN, Richardson DJ. 2007. Characterization of Shewanella oneidensis MtrC: a cell-surface decaheme cytochrome involved in respiratory electron transport to extracellular electron acceptors. J Biol Inorg Chem 12:1083–1094. doi:10.1007/s00775-007-0278-y 17701062

[76] Beblawy S, Bursac T, Paquete C, Louro R, Clarke TA, Gescher J. 2018. Extracellular reduction of solid electron acceptors by Shewanella oneidensis. Mol Microbiol 109:571–583. doi:10.1111/mmi.14067 29995975

[77] Grim CJ, Kothary MH, Gopinath G, Jarvis KG, Beaubrun J-G, McClelland M, Tall BD, Franco AA. 2012. Identification and characterization of cronobacter iron acquisition systems. Appl Environ Microbiol 78:6035–6050. doi:10.1128/AEM.01457-12 22706064 PMC3416605

[78] Wang Y, Ling N, Wang Y, Ou D, Liang Z, Li G, Zhao H, Ye Y. 2024. Effect of ferric ions on cronobacter sakazakii growth, biofilm formation, and swarming motility. Int J Food Microbiol 408:110418. doi:10.1016/j.ijfoodmicro.2023.110418 37857020

[79] Digel L, Bonné R, Aiyer K. 2024. Are all microbes electroactive? Cell Rep Phys Sci 5:102200. doi:10.1016/j.xcrp.2024.102200

[80] Dundas CM, Graham AJ, Romanovicz DK, Keitz BK. 2018. Extracellular electron transfer by Shewanella oneidensis controls palladium nanoparticle phenotype. ACS Synth Biol 7:2726–2736. doi:10.1021/acssynbio.8b00218 30396267

[81] Gao Y, Zhou Y, Ji X, Graham AJ, Dundas CM, Miniel Mahfoud IE, Tibbett BM, Tan B, Partipilo G, Dodabalapur A, Rivnay J, Keitz BK. 2024. A hybrid transistor with transcriptionally controlled computation and plasticity. Nat Commun 15:1598. doi:10.1038/s41467-024-45759-1 38383505 PMC10881478

[82] Zhu X, Wang K, Yan H, Liu C, Zhu X, Chen B. 2022. Microfluidics as an emerging platform for exploring soil environmental processes: a critical review. Environ Sci Technol 56:711–731. doi:10.1021/acs.est.1c03899 34985862

[83] Yuan S-J, He H, Sheng G-P, Chen J-J, Tong Z-H, Cheng Y-Y, Li W-W, Lin Z-Q, Zhang F, Yu H-Q. 2013. A photometric high-throughput method for identification of electrochemically active bacteria using A WO3 nanocluster probe. Sci Rep 3:1315. doi:10.1038/srep01315 23439110 PMC3581827

[84] Gralnick JA. 2012. On conducting electron traffic across the periplasm. Biochem Soc Trans 40:1178–1180. doi:10.1042/BST20120129 23176450

[85] Graham AJ, Dundas CM, Hillsley A, Kasprak DS, Rosales AM, Keitz BK. 2020. Genetic control of radical cross-linking in a semisynthetic hydrogel. ACS Biomater Sci Eng 6:1375–1386. doi:10.1021/acsbiomaterials.9b01773 33313392 PMC7725273

[86] Hofmann L, Hirsch M, Ruthstein S. 2021. Advances in understanding of the copper homeostasis in Pseudomonas aeruginosa. Int J Mol Sci 22:2050. doi:10.3390/ijms22042050 33669570 PMC7922089

[87] Virieux-Petit M, Hammer-Dedet F, Aujoulat F, Jumas-Bilak E, Romano-Bertrand S. 2022. From copper tolerance to resistance in Pseudomonas aeruginosa towards patho-adaptation and hospital success. Genes (Basel) 13:301. doi:10.3390/genes13020301 35205346 PMC8872213

[88] Patteson JB, Putz AT, Tao L, Simke WC, Bryant LH, Britt RD, Li B. 2021. Biosynthesis of fluopsin C, a copper-containing antibiotic from Pseudomonas aeruginosa Science 374:1005–1009. doi:10.1126/science.abj6749 34793213 PMC8939262

[89] Walter T, Klim J, Jurkowski M, Gawor J, Köhling I, Słodownik M, Zielenkiewicz U. 2020. Plasmidome of an environmental Acinetobacter lwoffi istrain originating from a former gold and arsenic mine. Plasmid 110:102505. doi:10.1016/j.plasmid.2020.102505 32380021

[90] Aweid O, Sundararajan S, Teferi A. 2016. Granulicatella adiacens prosthetic hip joint infection after dental treatment. JMM Case Rep 3:e005044. doi:10.1099/jmmcr.0.005044 28348763 PMC5330231

[91] Christensen JJ, Facklam RR. 2001. Granulicatella and Abiotrophia species from human clinical specimens. J Clin Microbiol 39:3520–3523. doi:10.1128/JCM.39.10.3520-3523.2001 11574566 PMC88382

[92] Mohapatra RK, Parhi PK, Thatoi H, Panda CR. 2017. Bioreduction of hexavalent chromium by Exiguobacterium indicum strain MW1 isolated from marine water of Paradip Port, Odisha, India. Chem Ecol 33:114–130. doi:10.1080/02757540.2016.1275586

[93] Chen B-Y, Hsueh C-C, Chen W-M, Li W-D. 2011. Exploring decolorization and halotolerance characteristics by indigenous acclimatized bacteria: chemical structure of azo dyes and dose–response assessment. J Taiwan Inst Chem Eng 42:816–825. doi:10.1016/j.jtice.2011.02.008

[94] Wu Y, Xiao X, Xu C, Cao D, Du D. 2013. Decolorization and detoxification of a sulfonated triphenylmethane dye aniline blue by Shewanella oneidensis MR-1 under anaerobic conditions. Appl Microbiol Biotechnol 97:7439–7446. doi:10.1007/s00253-012-4476-3 23053116

[95] Cai PJ, Xiao X, He YR, Li WW, Chu J, Wu C, He MX, Zhang Z, Sheng GP, Lam MHW, Xu F, Yu HQ. 2012. Anaerobic biodecolorization mechanism of methyl orange by Shewanella oneidensis MR-1. Appl Microbiol Biotechnol 93:1769–1776. doi:10.1007/s00253-011-3508-8 21808969

[96] Coursolle D, Baron DB, Bond DR, Gralnick JA. 2010. The Mtr respiratory pathway is essential for reducing flavins and electrodes in Shewanella oneidensis. J Bacteriol 192:467–474. doi:10.1128/JB.00925-09 19897659 PMC2805334

[97] Edwards MJ, White GF, Norman M, Tome-Fernandez A, Ainsworth E, Shi L, Fredrickson JK, Zachara JM, Butt JN, Richardson DJ, Clarke TA. 2015. Redox linked flavin sites in extracellular decaheme proteins involved in microbe-mineral electron transfer. Sci Rep 5:11677. doi:10.1038/srep11677 26126857 PMC4486940

[98] Larsen I, Little B, Nealson KH, Ray R, Stone A, Tian J. 1998. Manganite reduction by Shewanella putrefaciens MR-4. A M 83:1564–1572. doi:10.2138/am-1998-11-1244

[99] White GF, Edwards MJ, Gomez-Perez L, Richardson DJ, Butt JN, Clarke TA. 2016. Mechanisms of bacterial extracellular electron exchange. Vol. 68. Elsevier Ltd. 10.1016/bs.ampbs.2016.02.002 27134022

[100] Summers ZM, Fogarty HE, Leang C, Franks AE, Malvankar NS, Lovley DR. 2010. Direct exchange of electrons within aggregates of an evolved syntrophic coculture of anaerobic bacteria. Science 330:1413–1415. doi:10.1126/science.1196526 21127257

[101] Rizk AA, Komazin G, Maybin M, Hoque N, Weinert E, Meredith TC. 2023. The extracellular electron transport pathway reduces copper for sensing by the CopRS two-component system under anaerobic conditions in Listeria monocytogenes. J Bacteriol 205:e0039122. doi:10.1128/jb.00391-22 36622231 PMC9879103

[102] Komazin G, Rizk AA, Armbruster KM, Bonnell VA, Llinás M, Meredith TC. 2023. A copper-responsive two-component system governs lipoprotein remodeling in Listeria monocytogenes. J Bacteriol 205:e0039022. doi:10.1128/jb.00390-22 36622228 PMC9879112

[103] Mevers E, Su L, Pishchany G, Baruch M, Cornejo J, Hobert E, Dimise E, Ajo-Franklin CM, Clardy J. 2019. An elusive electron shuttle from a facultative anaerobe. Elife 8:1–15. doi:10.7554/eLife.48054 PMC6687433 31232690

[104] Wang Y, Kern SE, Newman DK. 2010. Endogenous phenazine antibiotics promote anaerobic survival of Pseudomonas aeruginosa via extracellular electron transfer. J Bacteriol 192:365–369. doi:10.1128/JB.01188-09 19880596 PMC2798253

[105] Hederstedt L, Gorton L, Pankratova G. 2020. Two routes for extracellular electron transfer in Enterococcus faecalis. J Bacteriol 202:1–9. doi:10.1128/JB.00725-19 PMC7167473 31932308

[106] Pankratova G, Hederstedt L, Gorton L. 2019. Extracellular electron transfer features of Gram-positive bacteria. Anal Chim Acta 1076:32–47. doi:10.1016/j.aca.2019.05.007 31203962

[107] Naradasu D, Miran W, Okamoto A. 2024. Electrochemical characterization of two gut microbial strains cooperatively promoting multiple sclerosis pathogenesis. Microorganisms 12:257. doi:10.3390/microorganisms12020257 38399661 PMC10892914

[108] Ishii S, Suzuki S, Tenney A, Nealson KH, Bretschger O. 2018. Comparative metatranscriptomics reveals extracellular electron transfer pathways conferring microbial adaptivity to surface redox potential changes. ISME J 12:2844–2863. doi:10.1038/s41396-018-0238-2 30050163 PMC6246609

[109] Bolyen E, Rideout JR, Dillon MR, Bokulich NA, Abnet CC, Al-Ghalith GA, Alexander H, Alm EJ, Arumugam M, Asnicar F, et al.. 2019. Reproducible, interactive, scalable and extensible microbiome data science using QIIME 2. Nat Biotechnol 37:852–857. doi:10.1038/s41587-019-0209-9 31341288 PMC7015180

[110] Oksanen, J, Simpson, GL, Blanchet, FG, et al.. 2022. Vegan: Community Ecology Package

[111] Wickham H. 2016. Ggplot2; use R! Springer International Publishing, Cham.

[112] Hashem A. 2019. Microbial fuel cell (MFC) application for generation of electricity from dumping rubbish and identification of potential electrogenic bacteria. AIB 2:1–8. doi:10.24966/AIB-5665/100010

